# Azole–Flavonoid Hybrids as Emerging Anticancer Agents: A Bioactivity-Focused Review

**DOI:** 10.3390/ph19020338

**Published:** 2026-02-20

**Authors:** Mihaela Lipovanu, Anca Miron, Nina Filip, Cristina Elena Horhogea, Ana Clara Aprotosoaie

**Affiliations:** 1 Grigore T. Popa University of Medicine and Pharmacy Iasi, 16, Universitatii Street, 700115 Iasi, Romania; 2 Ion Ionescu de la Brad Iasi University of Life Sciences, 3, Mihail Sadoveanu Alley, 700489 Iasi, Romania

**Keywords:** azoles, flavonoids, molecular hybridization, anticancer activity, leukemia cells, breast cancer cells, selective cytotoxicity, structure–activity relationship

## Abstract

Despite notable progress in drug discovery, cancer treatment remains hindered by limited therapeutic efficacy, poor target specificity, adverse effects, and the development of drug resistance. Molecular hybridization, which integrates two or more bioactive entities into a single molecule, has shown considerable potential to overcome these limitations. Since both azoles and flavonoids have demonstrated anticancer potential, extensive studies have been undertaken to combine the two entities and enhance the bioactivity of the resulting hybrids. In this context, numerous azole–flavonoid hybrids have been synthesized and investigated for their anticancer potential. This review provides an overview of the azole–flavonoid hybrids that are promising candidates for novel anticancer drug development, highlighting their superior antitumor potency compared to reference drugs, multitarget activity, tumor-selective cytotoxicity, efficacy against drug-resistant tumor cells, and structure–activity relationships. The review covers 250 hybrids, primarily triazole–chalcone hybrids but also triazole–flavone, flavanone, flavonol, and isoflavone hybrids, as well as other azole–flavonoid hybrids (imidazole–, pyrazole–, isoxazole–, and thiazole–flavonoid hybrids).

## 1. Introduction

As the second leading cause of mortality worldwide, cancer imposes a substantial strain on the healthcare system [1]. Despite significant advances in drug development, cancer treatment continues to face major limitations, including reduced efficacy, poor selectivity, severe side effects, and the emergence of drug resistance. These challenges highlight the need for innovative strategies to develop novel therapeutic agents capable of overcoming such drawbacks. In this respect, molecular hybridization, which combines two or more bioactive molecules or their pharmacophoric moieties into a single hybrid molecule, has proven to be a powerful approach [2]. The presence of two or more components that interact with different biological targets provides the novel hybrid entity with enhanced efficacy, improved safety, and reduced propensity to induce drug resistance [3,4,5]. Therefore, extensive research has been conducted to develop novel hybrid molecules with promising therapeutic potential. Among them, azole–flavonoid hybrids have gained considerable attention owing to their potent anticancer activity.

**Figure 1:**
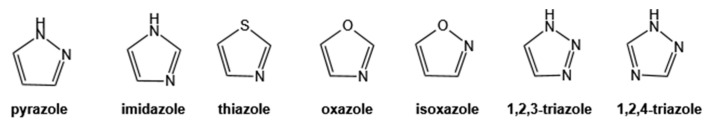
The basic ring scaffolds of azoles.

Azoles are electron-rich, five-membered nitrogen-containing heterocycles that include at least one additional heteroatom, such as nitrogen, sulfur, or oxygen (Figure 1) [1,6]. Their structural features facilitate noncovalent interactions (van der Waals forces, hydrogen bonds, coordination bonds, electrostatic interactions, and hydrophobic interactions) with biological targets, which account for their prominent role in drug discovery. Numerous commercially available drugs used to treat a wide range of diseases, including cancer, are azole derivatives. Dasatinib is used to treat chronic myeloid leukemia, while dacarbazine is indicated in Hodgkin’s lymphoma and malignant melanoma [1]. Moreover, azole-based compounds have demonstrated anticancer activity across various cancer cells, emphasizing the potential of the azole scaffold in the development of anticancer drugs [7].

**Figure 2:**
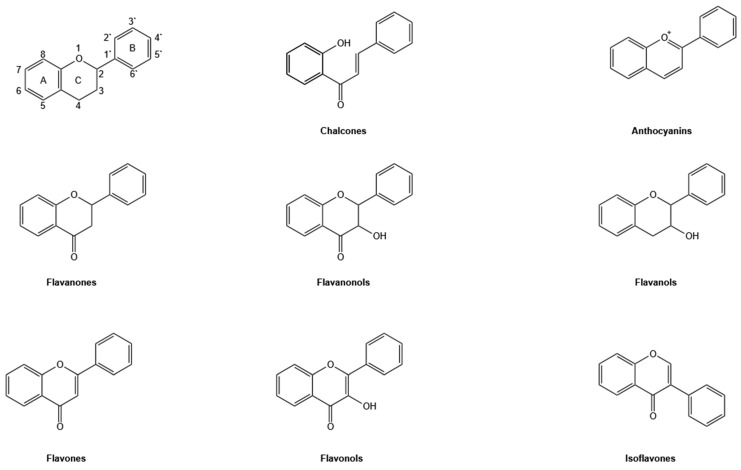
The basic structure of flavonoid subclasses.

Flavonoids are plant-derived specialized metabolites widely found in nature. Depending on the structural features of their basic C6-C3-C6 skeleton, flavonoids can be divided into several subclasses: chalcones, flavanones, flavanonols, flavones, flavonols, flavanols, anthocyanins, and isoflavones (Figure 2). They are essential for various plant processes, including growth, pollination, allelopathy, and protection against biotic and abiotic stresses, and have multiple applications in pharmaceuticals, nutraceuticals, and cosmetics due to their health-promoting effects [8,9]. Flavonoids offer significant health benefits for humans, as evidenced by numerous studies, including clinical trials. They possess a wide range of biological activities such as antioxidant, antimicrobial, antiviral, anti-inflammatory, anticancer, antimutagenic, antiallergic, antidiabetic, cardioprotective, and neuroprotective effects [10,11,12,13]. The anticancer potential of flavonoids is well-documented. Flavonoids modulate tumor survival, proliferation, and metastasis through multiple mechanisms, including the induction of cell death through apoptosis, necrosis, and autophagy, cell cycle arrest, inhibition of tumor angiogenesis and migration, and immunomodulation. These effects are achieved by acting on multiple targets, including proteins, enzymes, and signaling pathways. Moreover, certain flavonoids (apigenin, quercetin, genistein, daidzein, flavopiridol) have progressed to clinical trials for the development of antitumor agents [14]. In metastatic colorectal cancer patients, oral administration of genistein, the major soy isoflavone, in combination with folinic acid, 5-fluorouracil, and oxaliplatin (FOLFOX) or FOLFOX-bevacizumab, resulted in improved efficacy according to clinical trial endpoints (cycle 6 response rate, best overall response rate, and median progression-free survival) in a phase I/II pilot study [15]. In phase II clinical trials, flavopiridol (alvocidib) did not produce major tumor responses as a single agent in cancer patients and could not be administered safely in combination with docetaxel due to adverse effects [16]. But in patients with high-risk acute myeloid leukemia (phase II study), induction treatment with flavopiridol, cytarabine, and mitoxantrone (FLAM) was associated with higher complete remission rates compared with cytarabine and daunorubicin (70% vs. 46%), without an increase in overall toxicity [17].

The hybridization of azole and flavonoid moieties may synergistically enhance anticancer potency. Consequently, extensive research has been conducted to synthesize such hybrids and optimize their bioactivity. This review aims to summarize azole–flavonoid hybrids exhibiting notable anticancer potential and their relevance for the development of novel anticancer agents, addressing anticancer activity and structure–activity relationship (SAR), without covering synthesis or characterization methods. Although several reviews have discussed chalcone hybrids with anticancer activity [18], flavonoid–triazole hybrids, flavone hybrids, and natural products–isoxazole hybrids exhibiting diverse bioactivities [19,20,21], a focused review dedicated to azole–flavonoid hybrids across multiple azole and flavonoid subclasses, emphasizing their anticancer activity, is still lacking. The present review fills this gap by surveying azole–flavonoid hybrids that represent promising candidates for anticancer drug development. It covers 250 hybrids (127 triazole–flavonoid, 57 imidazole–flavonoid, 28 pyrazole–flavonoid, 15 isoxazole–flavonoid, and 19 thiazole–flavonoid hybrids), highlighting underrepresented classes (isoxazole–flavonoid and thiazole–flavonoid hybrids) that have received limited attention in previous reviews. Thus, this review offers a comprehensive perspective on anticancer azole–flavonoid hybrids.

## 2. Triazole–Flavonoid Hybrids

The triazole ring is a five-membered heterocycle containing three nitrogen atoms; two isomers exist (1,2,3- and 1,2,4-triazole), each exhibiting two tautomeric forms (1H- and 2H-1,2,3-triazole, and 1H- and 4H-1,2,4-triazole, respectively). The triazole moiety can act as both a hydrogen bond donor and acceptor and can participate in various noncovalent interactions with biological molecules, including hydrogen bonding, dipole–dipole, and stacking interactions. Consequently, triazole derivatives possess a wide range of pharmacological properties, including anticancer activity. Several commercially available drugs used to treat various malignancies incorporate a triazole scaffold, including anastrozole, letrozole, tucatinib, talazoparib, and selinexor [22,23].

The triazole scaffold has been reported to interact with well-known targets in anticancer therapy, such as tyrosine kinase, aromatase, microtubule, bromodomain and extraterminal (BET) and poly (ADP-ribose) polymerase (PARP) proteins. Combining the triazole scaffold with flavonoid skeletons (chalcone, flavanone, flavone, flavonol, and isoflavone) has yielded hybrids with remarkable cytotoxic activity against various cancer cell lines [22]. More than half of the hybrids discussed in this review are triazole–flavonoid hybrids, reflecting their higher prevalence in the literature compared with other azole–flavonoid hybrids.

### 2.1. Triazole–Chalcone Hybrids

**Figure 3:**
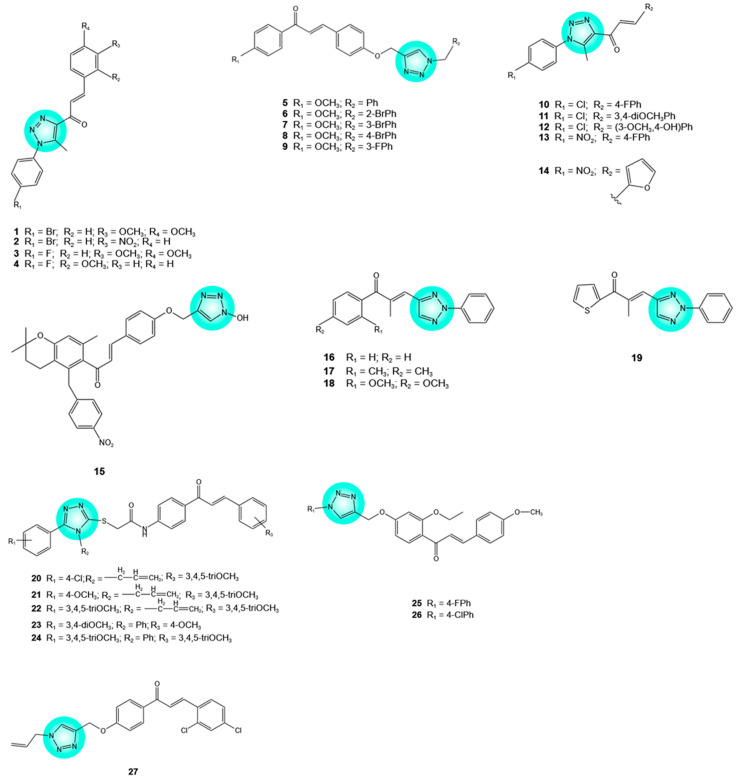

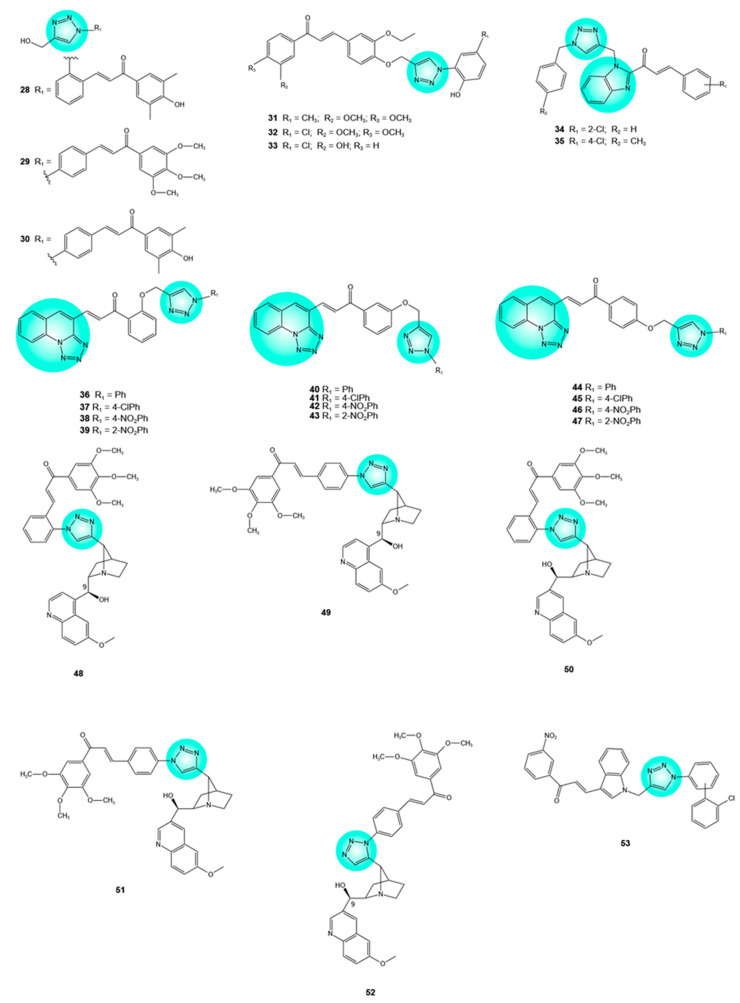

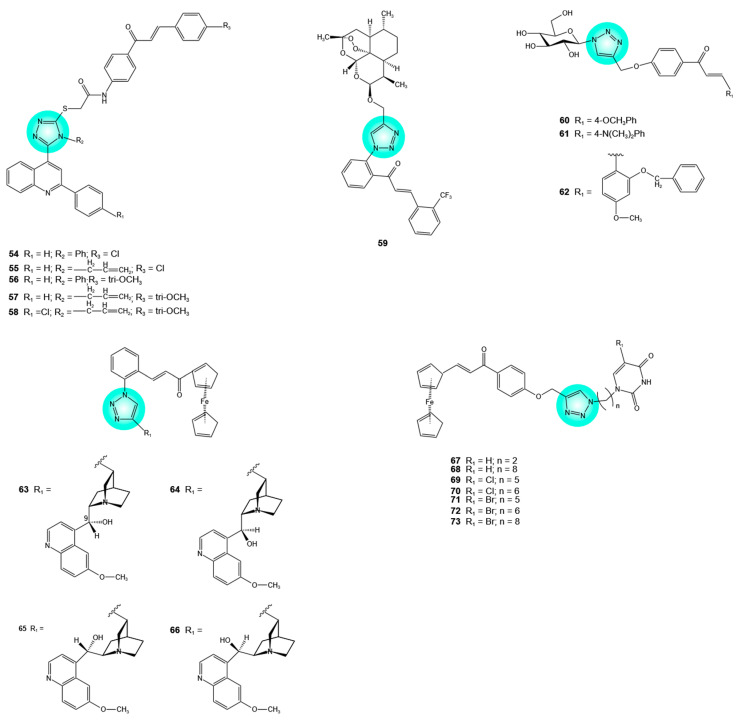
Triazole–chalcone hybrids with antitumor activity.

Triazole–chalcone hybrids have drawn particular interest because of their potent and selective anticancer activity. Thus, 1,2,3-triazole–chalcone hybrids **1**–**4** (Figure 3) inhibited the growth of RPMI-8226 leukemia cells with the half-maximal inhibitory concentration (IC_50_) values of 0.54, 0.26, 5.5, and 1.62 μM, respectively, being more active than the reference drug staurosporine (IC_50_ = 8.26 μM). Hybrid **2** was the most effective (almost 32-fold more active than staurosporine). It inhibited leukemia cell growth by inducing apoptosis through upregulation of genes encoding the pro-apoptotic B-cell lymphoma 2 (Bcl-2) associated X protein (Bax) and caspase-3 and-9 (*BAX*, *CASP3*, and *CASP9*, respectively), as well as causing cell cycle arrest at the S phase. In silico docking studies showed that all four hybrids interacted with the PARP-1 binding site through hydrogen bonding and hydrophobic interactions. The presence of a bromophenyl group at N-1 of the triazole unit and a *meta*-methoxy group on the enone-linked phenyl ring appears to have a positive impact on the activity. Moreover, hybrid **2** was highly selective for leukemia cells (IC_50_ = 3.17 μM against NCI-BL-2171 healthy human B lymphocytes, selectivity index = 12.19). In contrast, the other compounds and staurosporine exhibited higher toxicity against normal cells, with selectivity indices of 1.16–5.95 and 1.49, respectively [24].

Other 1,2,3-triazole–chalcone hybrids **5**–**9** (Figure 3) exhibited good activity (IC_50_ = 4–85 μM) against various human cancer cell lines, including MCF-7 (breast cancer), MIA-PaCa-2 (pancreatic cancer), A549 (non-small-cell lung cancer), and HepG2 (hepatocellular carcinoma), while being less active toward fR2 normal epithelial cells (IC_50_ > 100 μM). Hybrid **5** showed more pronounced inhibitory activity on MIA-PaCa-2 cells than the reference drug PI-103 (IC_50_ = 4 vs. 6 μM). In these cells, it induced apoptosis through both extrinsic and intrinsic pathways, as evidenced by the loss of mitochondrial membrane potential, the activation of pro-caspase-8 and -9, and the cleavage of caspase-3 and PARP-1. Additionally, it induced G2/S arrest. With respect to the relationship between chemical structure and biological activity, bromination and fluorination of the benzyl group at N-1 of the triazole ring resulted in decreased anticancer activity [25].

The hybridization between the chalcone scaffold of flavokawain B and 1,2,3-triazole unit afforded hybrids **10**–**14** (Figure 3), which were active against a broad spectrum of cancer cells. Among them, hybrid **11** was the most effective, exhibiting IC_50_ values in the nanomolar range (0.24–0.96 μM) against RPMI-8226, SR, K-562 (leukemia), M14 (melanoma), MCF-7 (breast), and HCT-116 (colon) cancer cells. It showed higher IC_50_ values (17.15–30.09 μM) over the corresponding normal cells, with selectivity index values above 30, indicating selective cytotoxicity against cancer cells. In RPMI-8226 leukemia cells, hybrid **11** promoted mitochondrial-mediated apoptosis (induction of oxidative stress, increase in Bax/Bcl-2 ratio, and activation of caspase-3, -7, and -9) and cell cycle arrest at the G2/M phase. The 3,4-dimethoxyphenyl chalcone moiety contained in hybrid **11** appears to play an important role in its potency and selectivity against tumor cells. Other hybrids also displayed IC_50_ values in the nanomolar range against the leukemia cell lines RPMI-8226 (**10**, **13**), CCRF-CEM (**12**, **14**), and HL (60)-TB (**12**), and showed high selectivity toward these cells [26].

Another hybrid exhibiting activity against multiple tumor cell lines is compound **15** (Figure 3). The latter exhibited significant antiproliferative activity (IC_50_ < 30 μM) in MCF-7 (breast cancer), DU-145 (prostate cancer), and IMR-32 (neuroblastoma) cells, being more potent than doxorubicin against IMR-32 cells (IC_50_ = 17.34 vs. 44.4 μM). In silico docking studies suggested that binding to DNA topoisomerase IIα (Topo IIα) may represent a potential mechanism contributing to the observed tumor cell toxicity [27].

1,2,3-Triazole–chalcone hybrids with activity against prostate cancer cells were also synthesized. Hybrids **16**–**19** (Figure 3) significantly reduced the viability of PC-3 prostate cancer cells (IC_50_ = 28.55, 15.64, and 25.56 μM, respectively). According to computational chemistry studies, the partial positive charge sites per unit of surface area exposed to the cell environment might be relevant for the observed activity [28].

1,2,4-Triazole–chalcone hybrids **20**–**24** (Figure 3) exhibited high cytotoxicity against A549 (non-small-cell lung cancer) cells (IC_50_ = 6.06, 4.4, 7.55, 16.04, and 8.04 μM, respectively), whereas cisplatin (reference drug) showed an IC_50_ value of 15.3 μM. With regard to the cytotoxicity of these triazole–chalcone hybrids, it is clear that allyl triazoles (**20**–**22**) were more active than phenyl triazoles (**23** and **24**). For the former, *para*-substitution on the phenyl ring with chloro or methoxy groups increased cytotoxicity, whereas, in the case of phenyl triazoles, 3, 4, 5-trimethoxy-substitution was crucial for bioactivity. The triazole–chalcone hybrids induced caspase-3-dependent apoptosis in A549 cells via both extrinsic (activation of caspase-8) and intrinsic (release of cytochrome *c* from mitochondria, activation of BAX and caspase-9) pathways [29]. The other two hybrids (**25** and **26**) (Figure 3) reduced the viability of A549 cells with IC_50_ values of 62.51 and 75.41 μM, respectively. Both hybrids contained a methoxy group on the chalcone B ring and a *para*-halophenyl substituent at N-1 of the triazole unit (*para*-fluoro and *para*-chloro-phenyl, respectively) [19,30].

The 1,2,3-triazole–chalcone hybrid **27** (Figure 3) is a promising lead compound for the development of drugs to treat liver cancer. The hybrid demonstrated antiproliferative activity against various liver cancer cells and was more active than the positive control, 5-fluorouracil (IC_50_ values of 0.9 vs. 9.2, 2.7 vs. 10.3, 6.2 vs. 15.1, and 4.6 vs. 17.9 μM against HepG2, SNU-423, SMMC7221, and SNU-398 cells, respectively). Hybrid **27** inhibited the growth, proliferation, and migration of HepG2 cells. The anti-migration effects were associated with the downregulation of upstream transcription factors Snail and Slug, N-cadherin, and vimentin and the upregulation of E-cadherin. N-cadherin, E-cadherin, and vimentin are key migration-related markers. Hybrid **27** also inhibited tubulin polymerization in HepG2 cells. Moreover, in a HepG2 xenograft mouse model, administration of hybrid **27** (70 mg/kg for 21 days) reduced tumor weights by 60.03% with no signs of toxicity [19,31].

1,2,3-Triazole–chalcone hybrids with promising activity against breast cancer cell lines were also reported. Hybrids **28**–**30** (Figure 3) showed promising cytotoxic activity against estrogen receptor-positive (MCF-7) and triple-negative (MDA-MB-231) breast cancer cells, with IC_50_ values ranging from 2.51 to 15.07 and 4.40 to 11.11 μM, respectively. Notably, hybrid **28** exhibited higher activity than cisplatin (IC_50_ = 2.51 vs. 5.35 and 4.40 vs. 26.15 μM, respectively) [19,32]. Similarly, hybrids **31**–**33** (Figure 3) exhibited significant cytotoxicity against MCF-7 and MDA-MD-231 cells, with hybrid **32** being more active than cisplatin (IC_50_ = 0.02 vs. 1.28 μM and 0.31 vs. 7.34 μM, respectively). The 3,4-dimethoxybenzene A ring in the chalcone moiety and a *meta*-chloro substituent on the benzene ring significantly increased antitumor activity. In addition, the three hybrids were less cytotoxic toward non-tumorigenic MCF10A epithelial cells, with hybrid **32** showing the lowest cytotoxicity (selectivity indices of 6964.5 and 449.32 against MCF-7 and MDA-MD-231 cells, respectively, compared with 19.46 and 3.39 for cisplatin) [19,33].

More complex structures, namely tri-pharmacophore molecular hybrids incorporating triazole and chalcone moieties, have been reported to exhibit excellent cytotoxic activity against various tumor cell lines. 1,2,3-Triazole–benzimidazole–chalcone hybrids **34** and **35** (Figure 3) had significant effects in PC-3 prostate cancer cells (IC_50_ = 10.7 and 5.64 μM, respectively); hybrid **34** was also active against T47-D and MDA-MB-231 breast cancer cells (IC_50_ = 6.23 and 5.89 μM, respectively). When the activity of these two hybrids was compared to that of other compounds in the same series, it was noticed that the presence of the benzyl group at N-1 of the 1,2,3-triazole unit and a chloro substitution at the chalcone moiety are essential for cytotoxic activity [19,34].

1,2,3-Triazole–tetrazoloquinoline–chalcone hybrids **36**–**47** (Figure 3) displayed higher cytotoxicity than doxorubicin against SiHa cervical squamous carcinoma cells (concentration causing 50% growth inhibition (GI_50_) = 0.51–2.01 vs. 2.31 μM), MDA-MB-231 breast cancer cells (GI_50_ = 0.38–1.01 vs. 1.15 μM), and PANC-1 pancreatic cancer cells (GI_50_ = 1.16–2.67 vs. 3.10 μM) [35].

Cinchona alkaloids (quinine, quinidine) do not possess intrinsic antitumor activity, but some of them can enhance the activity of anticancer drugs. Moreover, hybrids including two cinchona alkaloid fragments in C2-symmetric arrangements showed cytotoxic and cytostatic effects against various human cancer cells. Based on these considerations, 23 quinine/quinidine–chalcone hybrids linked by 1,4- or 1,5-disubstituted-1,2,3-triazole rings have been synthesized and evaluated for in vitro antitumor activity. Among 1,4-disubstituted-triazole-linked hybrids, those featuring a 3,4,5-trimethoxybenzoyl group within the chalcone moiety (hybrids **48**–**51**) (Figure 3) showed the most promising cytotoxic activity (IC_50_ = 0.87–6.59 μM) against pancreatic cancer (PANC-1), colon cancer (COLO-205), non-small-cell lung cancer (EBC-1), and melanoma (A-2058) cells. With regards to 1,5-disubstituted-triazole-linked hybrids, hybrid **52** (Figure 3), containing an epiquinidine scaffold and a 3,4,5-trimethoxybenzoyl group in the chalcone structure, was the most active (IC_50_ = 0.55–2.26 μM). For these hybrids, epimerization at C9 enhanced their cytotoxicity. In hybrid **52,** the full rotation of 1,5-disubstituted-triazole is restricted by the chalcone and quinuclidine moieties, providing the structural rigidity required for selective binding to biological targets and, consequently, high bioactivity. In PANC-1 cells, hybrid **52** induced cell cycle arrest at the subG1 (apoptotic effect), S, and G2/M phases [36].

The anticancer properties of the indole scaffold are well-known, and many natural (vinblastine, vincristine), semisynthetic (vinorelbine), and synthetic (dacinostat, panobinostat, indibulin, bizelesin, MKC-1, NB-506) antitumor agents incorporate an indole moiety. Hybrid **53** (Figure 3), incorporating indole and chalcone moieties tethered with a 1,2,3-triazole linker, yielded IC_50_ values of 67.99 and 48.96 μg/mL against cervical and colorectal cancer cells (SiHa and SW620, respectively), with no notable cytotoxicity toward normal cells (HEK293 embryonic kidney cells) at the concentrations mentioned above. Spectroscopic and molecular docking studies revealed that hybrid **53** binds to DNA through noncovalent intercalation, involving the phenyl group and the triazole nitrogen [37].

1,2,4-Triazole tethered quinoline–chalcone hybrids **54**–**58** (Figure 3) were screened for their cytotoxic activity against pancreatic (PANC-1), breast (MCF-7), colon (HT-29), and lung (A549) cancer cells. The IC_50_ values ranged from 2.9 to 8.2 μM, whereas those of the positive control, doxorubicin, varied from 0.90 to 1.41 μM. Hybrid **55**, possessing an allyl-triazole moiety, was the most active (IC_50_ = 2.9, 3.3, 3.5, and 3.6 μM, respectively). Hybrids containing an allyl-triazole scaffold were more active than those bearing a phenyl-triazole moiety. Other structural features important for the cytotoxic activity included the chloro substitution on the 3-phenyl acryloyl ring, the 3,4,5-trimethoxy group on the same ring, and the unsubstituted phenyl ring of the quinoline unit [38]. The epidermal growth factor receptor (EGFR) is a major regulator of intracellular signaling pathways that control key cellular processes, including survival, growth, differentiation, proliferation, and migration, and is involved in cancer development [39]. Hybrids **54**–**58** inhibited EGFR and BRAF^V600E^ oncogenic kinases with IC_50_ values of 1.3–4.8 and 1.1–6.9 μM, respectively, with **55** and **58** being the most active hybrids. According to molecular docking studies, hybrids **54**–**58** fitted into the active sites of the two kinases [38].

Artemisinin, the antimalarial compound isolated from *Artemisia annua* L., is also endowed with anticancer activity, mainly attributed to its endoperoxide bond. The hybridization of artemisinin and chalcone moieties linked by 1,2,3-triazole generated derivatives with antiproliferative activity. Among these, hybrid **59** (Figure 3) is particularly noteworthy. It reduced the proliferation rate of squamous carcinoma cells (A431), erythromyeloblastoid leukemia (K562), prostate (PC-3), breast (MDA-MB-231), colon (COLO-205), and lung (A549) cancer cells with IC_50_ values of 7.16–28.21 μM, with no toxicity for HEK293 normal cells. A *para*-phenolic hydroxyl group on the benzene ring, along with an *ortho* trifluoromethyl substitution on the B ring of the chalcone moiety, significantly enhanced antiproliferative activity. The hybrid induced apoptosis and cell cycle arrest at the G2/M phase and increased oxidative stress in A431 and A549 cells. Artemisinin exhibited lower activity against all six cancer cell lines (IC_50_ > 39.03 μM), whereas doxorubicin exhibited IC_50_ values of 1.43–11.99 μM [40].

Three glycosyl–chalcone hybrids linked by 1,2,3-triazole **60**, **61**, and **62** (Figure 3) attracted attention due to their selective cytotoxicity against MDA-MB-468 (triple-negative) and MCF-7 breast cancer cells. Hybrid **61** was the most active against breast cancer cells, displaying concentrations causing 50% cell death (LD_50_) of 28 and 31 μM, respectively, while exhibiting the highest selectivity, with selectivity indices of 2.11 and 1.9, respectively. Etoposide and paclitaxel, used as positive controls, had LD_50_ and selectivity index values in the ranges of 27–38 μM and 0.95–1.29, respectively. In both cancer cells, hybrid **61** increased oxidative stress and mitochondrial permeability and induced DNA fragmentation, resulting in apoptotic cell death [41].

Ferrocene is a sandwich-type organometallic compound consisting of an iron center and two cyclopentadienyl ligands. In intracellular environments or in the presence of peroxidases, it can be oxidized to the ferrocenium cation, which exhibits significant cytotoxic effects. In addition, ferrocene is highly stable and can undergo diverse functionalization. Ferrocene derivatives exhibiting excellent cytotoxic activity against various cancer cell lines have been reported. These derivatives also include ferrocene analogues of flavonoids such as ferrocenyl aurones, ferrocenyl flavones, and ferrocenyl chalcones [42]. Ferrocenyl chalcones linked via 1,2,3-triazole to other bioactive entities (quinine/quinidine, uracil, tetrahydro-beta-carboline) **63**–**66** (Figure 3) yielded tri-pharmacophore hybrids with promising anticancer activity. Ferrocenyl chalcone–quinine/quinidine hybrids linked by 1,2,3-triazole were active against both sensitive and multidrug-resistant (MDR) non-small-cell lung carcinoma (NCI-H460 and NCI-H460/R, respectively), colorectal carcinoma (DLD1 and DLD1-TxR, respectively), and glioblastoma (U87 and U87-TxR, respectively) cells (IC_50_ = 1.60–10.71 μM). NCI-H460/R, a doxorubicin-resistant line, and DLD1-TxR and U87-TxR cells, paclitaxel-resistant lines, were generated through prolonged exposure of the parental cells to doxorubicin and paclitaxel, respectively [19,43]. All three resistant cancer cell lines express P-glycoprotein (P-gp) [44,45], an ATP-binding cassette (ABC) transporter that mediates drug efflux from cancer cells [46,47]. Hybrids **65** and **66**, containing a quinidine moiety, showed higher selectivity toward MDR cancer cells than hybrids **63** and **64**, which contain a quinine moiety. In MDR cancer cells (DLD1-TxR and U87-TxR), hybrids **65** and **66** increased oxidative stress, reduced mitochondrial membrane potential, induced apoptosis and necrosis, inhibited autophagy, and increased sensitivity to paclitaxel [19,43]. Seven ferrocenyl chalcones conjugated with uracil via a 1,2,3-triazole moiety and an alkyl chain **67**–**73** (Figure 3) reduced the proliferation of CCRF-CEM leukemia cells by approximately 70% after 72 h of treatment and exhibited lower cytotoxicity against LLC-PK1 normal kidney cells. The hybrids, having long alkyl chains (*n* = 5, 6, and 8), were tested at 50 μM [19,48].

### 2.2. Triazole–Flavanone Hybrids

**Figure 4:**
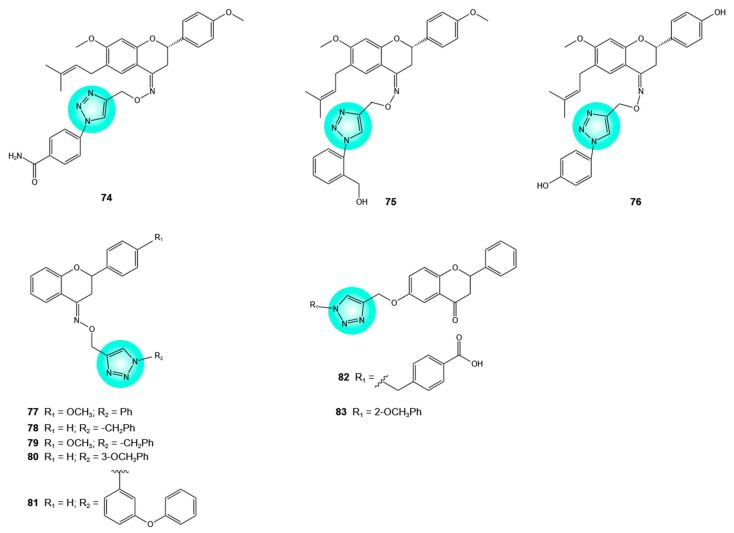
Triazole–flavanone hybrids with antitumor activity.

The hybridization between flavanones and 1,2,3-triazole has also yielded hybrids exhibiting remarkable antiproliferative effects in tumor cells. Bavachinin, a major flavanone in *Psoralea corylifolia* L. seeds, has demonstrated cytotoxic effects on various cancer cell lines. Among a series of synthesized 1,2,3-triazole-bavachinin hybrids, compound **74** (Figure 4) was highly active against lung (A549), prostate (PC-3), colon (HCT-116), and breast (MCF-7) cancer cells (IC_50_ = 7.72, 16.08, 7.13, and 11.67 μM, respectively). In HCT-116 cells, hybrid **74** reduced colony formation and cell migration, induced morphological changes, loss of mitochondrial membrane potential, and cleavage of PARP, a marker of apoptosis. At 50 μM, hybrid **75** (Figure 4) induced 100% growth inhibition in A549, PC-3, HCT-116, and MCF-7 cells, whereas hybrid **76** (Figure 4) caused growth inhibition ranging from 52% to 65%. In general, hybrids having electron-withdrawing groups were more active than those having electron-donating groups. The benzamide moiety in hybrid **74** contributes significantly to its antitumor activity [49].

Other 1,2,3-triazole–flavanone hybrids **77**–**81** (Figure 4) with good antiproliferative activity (IC_50_ = 5.4–32.9 μM) against HCT-15 colorectal, HeLa cervical, and NCI-H522 lung cancer cells, without affecting HEK293 normal cells, have been reported [50].

The discovery of Breakpoint Cluster Region–Abelson (BCR–ABL) kinase inhibitors such as imatinib, dasatinib, nilotinib, and bosutinib has significantly improved the management of chronic myeloid leukemia [51]. Two 1,2,3-triazole–flavanone hybrids **82** and **83** (Figure 4), with excellent BCR–ABL kinase inhibitory effects (IC_50_ = 364 and 275 nM, respectively), have been described. This kinase, an oncogenic fusion tyrosine kinase, is a hallmark of chronic myeloid leukemia. Hybrid **82** was found to interact with amino acid residues in the ATP-binding pocket of the kinase and to induce conformational changes. In silico studies predicted favorable passive permeation of hybrid **83** across the blood–brain barrier and a P-gp-mediated transport for hybrid **82** [19,52].

### 2.3. Triazole–Flavone Hybrids

**Figure 5:**
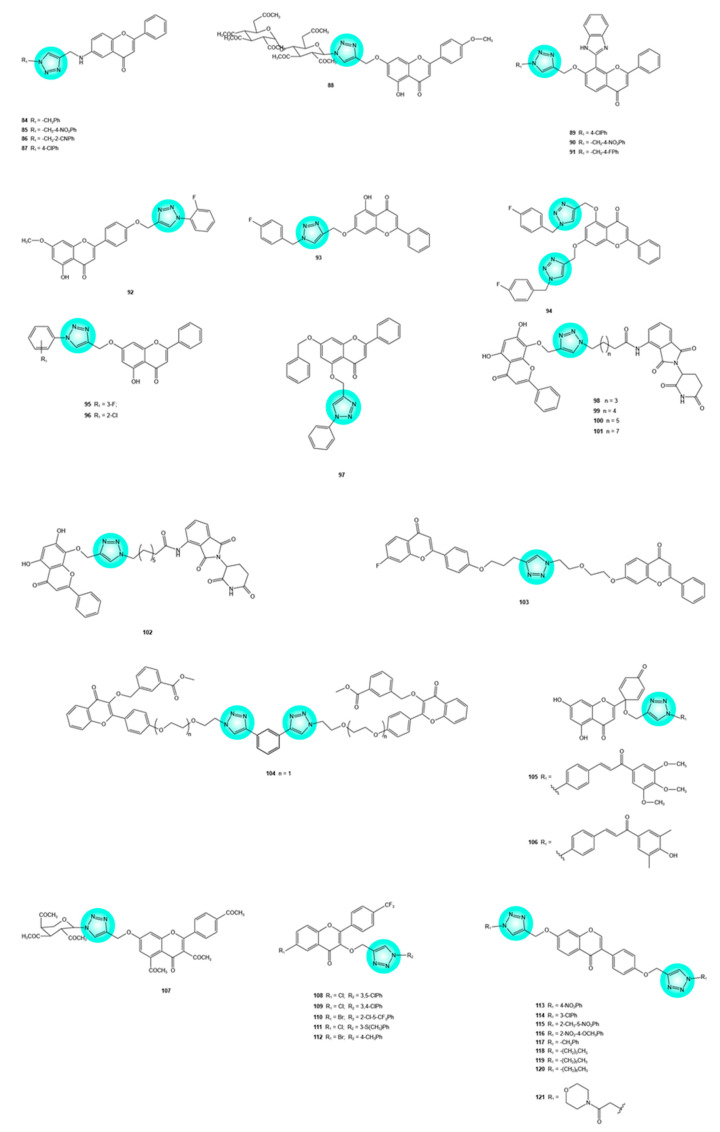

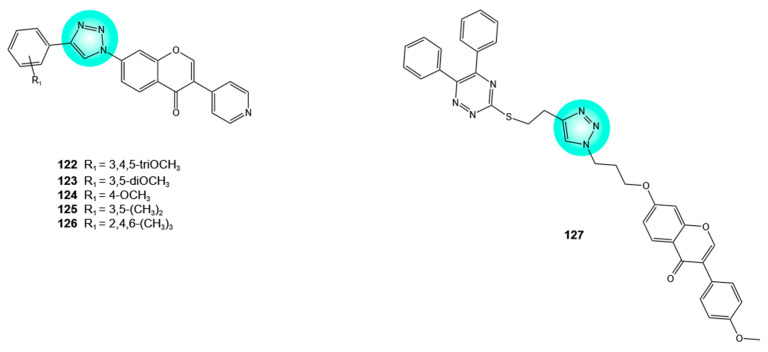
Other azole–flavonoid hybrids with antitumor activity.

Numerous 1,2,3-triazole–flavone hybrids have been synthesized and tested for their antitumor potential. Hybrids **84**–**87** (Figure 5) demonstrated promising activity against MDA-MB-231 triple-negative breast (**84**–**86**) and IMR-32 neuroblastoma (**86**, **87**) cells with GI_50_ values below 0.7 μM. Hybrid **85** (GI_50_ < 0.01 μM) was more potent than doxorubicin (GI_50_ = 0.085 μM) and paclitaxel (GI_50_ = 0.091 μM) against MDA-MB-231 cells. A structure–activity relationship analysis of the hybrids of this series revealed that an electron-withdrawing substituent on the benzene ring linked to the triazole moiety seems to be essential for the observed antitumor activity, whereas an alkyl chain or *meta* substitution on the same ring negatively impacts bioactivity [19,53]. 

Compound **88** (Figure 5), a 1,2,3-triazole–flavone glycoside hybrid, displayed higher activity than the positive control, cisplatin, against HeLa cervical cancer cells (IC_50_ = 14.67 vs. 21.30 μM) [19,54]. Hybrids **89**–**91** (Figure 5) were distinguished by their antiproliferative activity against MCF-7 cells (IC_50_ = 17.9, 14.2, and 19.1 μM, respectively), with two structural features being critical for bioactivity: an electron-withdrawing group at C-4 of the benzene ring and an unsubstituted flavone scaffold [19,55].

Hybrids containing an apigenin-7-methyl ether scaffold exhibited promising activity against ovarian cancer cells (SKOV3, OVCAR-3, and Caov-3). Among them, hybrid **92** (Figure 5) showed the most promising activity, displaying IC_50_ values of 10, 15, and 20 μM, respectively. In SKOV3 cells, the compound induced apoptosis. Several apoptosis-related effects, such as increase in oxidative stress, decrease in mitochondrial membrane potential, downregulation of Bcl-2 expression, and upregulation of Bax expression, were observed in SKOV3 cells exposed to hybrid **92** [19,56].

Chrysin, a flavone occurring in numerous edible plant species and known for its anticancer properties, has been used as a building block to synthesize hybrids with a triazole moiety. Hybrids **93** and **94** (Figure 5) demonstrated considerable activity against HeLa cervical cancer cells compared with cisplatin (IC_50_ = 1.909 and 0.7331 μM, respectively, vs. 12.26 μM). Hybrid **93**, a bis-hybrid compound, was also highly active against SiHa cervical cancer cells (IC_50_ = 1.352 μM vs. 5.305 μM for cisplatin) [57]. Hybrids **95** and **96** (Figure 5), synthesized in another study, were more active than 5-fluorouracil in reducing the proliferation of MGC-803 gastric cancer cells (IC_50_ = 18.40 and 5.92 μM vs. 30.52 μM) [58]. Another 1,2,3-triazole-chrysin hybrid **97** (Figure 5) demonstrated good activity (IC_50_ = 10.8–53.8 μM) against various cancer cells (PC3, PC3-PSMA, MCF-7, and UM-UC-3), with higher efficacy than doxorubicin on PC-3 and MCF-7 cells. The presence of a phenyl group at the 1,2,3-triazole unit and a benzyl group at C7 of the flavone moiety seems to be responsible for the anticancer potency [19,59].

Cyclin-dependent kinase 9 (CDK9) regulates the expression of anti-apoptotic proteins critical for cancer cell survival, and therefore, it has become an important therapeutic target in oncology. Conjugation of wogonin (CDK9 inhibitor) with pomalidomide (thalidomide derivative able to induce targeted proteolysis by recruiting cereblon, an E3 ubiquitin ligase substrate receptor) through 1,2,3-triazole and alkyl chain linkers afforded hybrids **98**–**101** (Figure 5). Hybrid **101** displayed an IC_50_ value of 17 μM against CDK9-rich MCF-7 cells, along with CDK9 degradation (IC_50_ = 523 nM), downregulation of the prosurvival protein Mcl-1, and induction of apoptosis [19,60].

Proteolysis Targeting Chimera (PROTAC) is an innovative technology that enables the selective degradation of target proteins, offering significant potential as a therapeutic approach for various diseases, including cancer. PROTAC uses a heterobifunctional molecule that simultaneously binds to the target protein and E3 ubiquitin ligase. The target protein is ubiquitinated, followed by recognition and degradation by the ubiquitin–proteasome system [61]. Triazole has been widely employed in the synthesis of PROTACs designed to degrade specific proteins, due to its significant advantages, including enhanced target protein degradation through stabilization of the PROTAC–target protein–E3 ubiquitin ligase ternary complex, as well as increased affinity of the PROTAC for the target protein [61]. Hybrid **102** (Figure 5), a PROTAC incorporating triazole and wogonin, has been reported to degrade CDK9 by 61.2% at 1 μM [60,61,62].

1,2,3-Triazole-linked flavone dimers represent an interesting class of hybrids with potential applications in reversing MDR in cancer cells via the inhibition of multidrug-resistance-associated protein 1 (MRP1) and breast cancer resistance protein (BCRP), both members of ABC transporters involved in MDR. Among the numerous synthesized hybrids, several exhibited particularly notable activity. Hybrid **103** (Figure 5) competitively inhibited MRP1, with a concentration producing 50% of the maximal effect (EC_50_) of 53 nM, by targeting its bipartite substrate-binding site; verapamil, a well-known MRP1 inhibitor, displayed a considerably higher EC_50_ value of 1950 nM [19,63]. Hybrid **104** (Figure 5) showed a remarkable BCRP inhibitory activity (EC_50_ = 1–2 nM), reversing drug resistance in BCRP-overexpressing cells [19,64]. Hybrids **105** and **106** (Figure 5), containing a protoflavone (protoapigenone) and a chalcone linked via 1,2,3-triazole, reduced the viability of MDA-MB-231 triple-negative breast cancer cells; hybrid **105** induced apoptotic cell death, as evidenced by subG1 phase arrest and an increase in caspase-3 activity [32].

### 2.4. Triazole–Flavonol Hybrids

Several 1,2,3-triazole–flavonol hybrids with promising antitumor activity have been described in the literature. Hybrid **107** (Figure 5) selectively reduced the proliferation of HeLa, HepG2, A549, MGC-803, and SCG-7901 cancer cells with IC_50_ values of 86.183, 88.149, 95.842, 82.992, and 83.421 μM, respectively, being more active than 5-fluorouracil (IC_50_ = 110.164, 110.013, 105.712, 95.172, and 96.011 μM, respectively). The hybrid efficiently inhibited human telomerase (IC_50_ < 50 μM), an enzyme associated with cancer cell proliferation. According to molecular simulation assays, hybrid **107** interacted with Lys437 and Asn421 of telomerase through hydrogen bonding, similar to BIBR1532, a telomerase-specific inhibitor [19,65].

Other 1,2,3-triazole–flavonol hybrids demonstrated high cytotoxicity (IC_50_ < 3 μM) against HCT-116 colon cancer cells (**108**–**110**) and OVCAR-3 ovarian cancer cells (**111** and **112**) (Figure 5). Structural features such as halogen groups on R_1_, a trifluoromethyl group on the benzene ring, and a substituted phenyl ring attached to the triazole moiety had a positive impact on cytotoxicity [19,66].

### 2.5. Triazole-Isoflavone Hybrids

Several triazole–isoflavone hybrids demonstrated promising activity for the development of future anticancer drugs. 1,2,3-Triazole-daidzein hybrids **113**–**121** (Figure 5), with excellent activity (GI_50_ = 0.14–0.88 μM) against A549 lung cancer, HeLa cervical cancer, and MDA-MB-231 triple-negative breast cancer cells, have been described [67].

1,2,3-Triazole-isoflavone hybrids **122**–**126** (Figure 5) demonstrated superior activity compared with etoposide against PC-3 and DU-145 prostate, MCF-7 breast, and A549 lung cancer cells (IC_50_ = 0.02–2.35 μM vs. 1.97–3.08 μM). Hybrid **122** exhibited the highest inhibitory activity against all four cancer cell lines, with IC_50_ values below 1 μM (nanomolar range). Similar to **122**, hybrid **123** showed nanomolar IC_50_ values, but only against three of the four cancer cell lines (PC-3, MCF-7, and A549). The substituents on the phenyl ring attached to the 1,2,3-triazole unit had a significant impact on bioactivity. Thus, hybrid **122** featured a 3,4,5-trimethoxy-substituted phenyl moiety, whereas hybrid **123** exhibited a 3,5-dimethoxy substitution pattern. Hybrid **124**, bearing a 4-methoxy group on the phenyl ring, was more active than hybrids **125** and **126** (3,5-dimethyl and 3,4,5-trimethyl substitution patterns on the phenyl ring, respectively) against all four cancer cell lines. Molecular docking studies demonstrated the ability of hybrids **122** and **123** to interact with the androgen receptor ligand-binding domain, using a selective androgen receptor modulator-bound structure (PDB ID: 3V49) and Abl-tyrosine kinase (PDB ID: 1IEP) [68]. The androgen receptor regulates survival, growth, metabolism, and differentiation in normal prostate epithelial cells as well as in prostate cancer cells and is therefore an important therapeutic target in this disease [69]. Abl-Tyrosine kinase is upregulated in various solid tumors, including those of the breast and lung. Hybrids **122** and **123** showed favorable docking scores and formed hydrogen bonds and hydrophobic interactions with amino acid residues in the active sites of both target proteins, supporting their potent activity against PC-3, DU-145, MCF-7, and A549 cancer cells. Quantitative estimation of drug-likeness indicated that all hybrids possess good oral bioavailability [68].

A 1,2,4-triazole-isoflavone hybrid **127** (Figure 5) has emerged as a compound of interest due to its selective cytotoxicity against PC-3 cancer cells, surpassing that of 5-fluorouracil (IC_50_ = 10.23 vs. 12.87 μM) [19,70].

## 3. Imidazole–Flavonoid Hybrids

Imidazole, a highly polar five-membered heterocycle with two nitrogen atoms, can engage in diverse molecular interactions, such as hydrogen bonding, π-π stacking, and van der Waals and cation-π interactions. Imidazoles demonstrated a remarkable anticancer potential mediated by interaction with multiple targets, including tubulin, tyrosine and serine–threonine kinases, G-quadruplexes, topoisomerases, minichromosomal maintenance proteins, histone deacetylase, and PARP, among others [71]. Furthermore, several imidazole-containing anticancer drugs, namely dacarbazine, nocodazole, bendamustine, veliparib, abemaciclib, glasdegib, liarozole, and tipifarnib, are currently available on the market [72]. Hybridization of imidazole and flavonoid moieties has generated agents with potent, multitarget, and synergistic anticancer effects.

**Figure 6:**
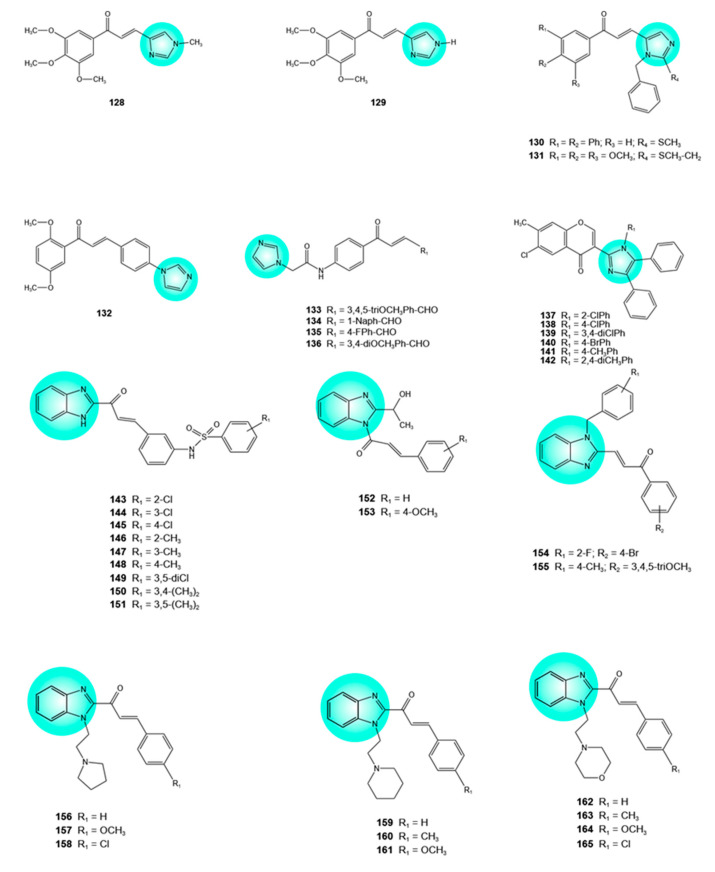

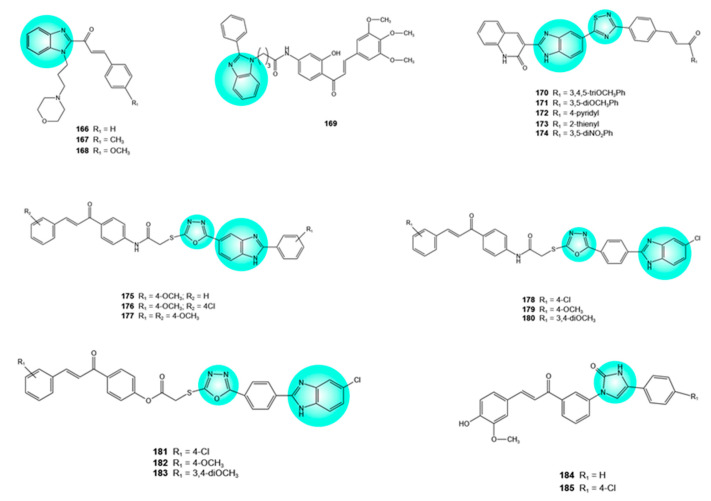
Imidazole–flavonoid hybrids with antitumor activity.

Imidazole–chalcone hybrids **128** and **129** (Figure 6) exhibited high antiproliferative activity against HT-29 colon cancer cells and MDA-MB-231 metastatic breast cancer cells (IC_50_ = 2.9, 4.8 and 5.0, 4.9 μM, respectively). N-1 methylation of imidazole clearly resulted in higher anticancer potency. It should be emphasized that hybrid **129**, the most active one, contained an N-methylurocanic moiety, which is also present in eleutherobin and sarcodyctyins, antitumor natural products isolated from soft coral [72,73].

Hybrids **130** and **131** (Figure 6) showed significant cytotoxicity against human breast cancer cells resistant to mitoxantrone (MCF-7/MX), with IC_50_ values of 14.94 and 20.2 μM, respectively. Both hybrids also exhibited notable cytotoxic effects against MCF-7 breast cancer (IC_50_ = 14.85 and 9.88 μM, respectively) and A549 lung cancer cells (IC_50_ = 11.7 and 7.05 μM, respectively). Among the tested cancer cell lines, A549 cells were the most sensitive to hybrids **130** and **131**. In these cells, the cytotoxic effects of the hybrids occurred via cell cycle arrest at the G2/M phase, the induction of apoptosis (cell accumulation in the subG1 phase), and the inhibition of tubulin polymerization. Molecular docking studies of the hybrid **131**, the more effective of the two hybrids, at the colchicine-binding site of tubulin, revealed interactions with various residues of the protein (hydrogen bond interactions with Ser178α and Ala316β, cation-π interaction with Asn258β, hydrophobic interactions with Glu183α, Thr224α, Lys254β, Asn101α, Val351β, Lys352β, and Leu248β). Hybrid **131** bears three methoxy groups on the A ring, a structural feature common to active tubulin polymerization inhibitors such as combretastatin A-4 [74,75].

Other imidazole–chalcone hybrids that inhibit tubulin polymerization and microtubule assembly have been reported. Thus, hybrid **132** (Figure 6) is promising, considering its strong activity against cervical cancer cell lines (HeLa, SiHa, C-33A) and low activity against H8 normal cells (IC_50_ = 2.28, 7.77, 4.20, and 47.51 μM, respectively). Cisplatin, a well-known antitumor agent, showed lower efficacy against the three cancer cell lines and higher toxicity toward the same normal cells (IC_50_ = 13.89, 18.90, 11.70, and 23.17 μM, respectively). Hybrid **132** exhibited significant cytotoxic activity (IC_50_ = 3.72 μM) against cisplatin-resistant cervical cancer cells (HeLa/DDP) and, moreover, reversed cisplatin resistance in these cells, as evidenced by the decreased resistance index upon coadministration with cisplatin. In HeLa and HeLa/DDP cells, hybrid **132** inhibited microtubule assembly, arrested cell cycle at the G2/M phase, induced apoptosis, and inhibited colony formation, cell migration and invasion. Inhibition of microtubule assembly was due to multiple interactions (carbon–hydrogen bond, π-σ, and π-alkyl interactions) between the imidazole, phenyl, and methoxy moieties of hybrid **132** and various amino acid residues in the colchicine-binding site of tubulin. In addition, in HeLa/DDP cells, hybrid **132** inhibited the activity of P-gp [76]. P-gp is found in various tissues and overexpressed in cancer cells, mediating the efflux of chemotherapeutic agents from cancer cells and contributing to the development of MDR [46,47]. Co-administration of chemotherapeutic agents with P-gp inhibitors has emerged as a promising strategy to overcome MDR [46,47]. In the case of hybrid **132**, the dimethoxy substitution on the phenyl ring was critical for the observed antitumor activity [76].

Aromatase, the enzyme responsible for converting androgens into estrogens, plays a key role in the development and progression of estrogen-dependent breast cancer, and aromatase inhibitors are therefore commonly used in its treatment. Four imidazole–chalcone hybrids **133**–**136** (Figure 6) have been reported to inhibit aromatase in vitro, with IC_50_ values of 2.76, 6.45, 4.82, and 3.00 μM, respectively, whereas letrozole, a drug used to treat estrogen-dependent breast cancer, exhibited an IC_50_ of 0.98 μM. The four hybrids were more effective than letrozole in reducing the viability of MCF-7 breast cancer cells (IC_50_ = 18.13, 21.71, 16.36, and 21.06 μM, respectively, vs. 30.39 μM). The presence of a methoxy group on the chalcone moiety was important for aromatase inhibition. All hybrids showed acceptable absorption, distribution, metabolism, and excretion (ADME) properties [77].

Imidazole–flavone hybrids **137**–**142** (Figure 6) reduced the proliferation of MCF-7 breast cancer, A549 lung cancer, and HepG2 liver cancer cells with IC_50_ values from 1.56 to 12.5 μM; the presence of electron-withdrawing groups was essential for good activity [78].

Similarly to imidazole, benzimidazole also exhibits anticancer activity, being one of the most promising scaffolds for antitumor drug development. This property derives from its strong ability to interact with proteins through noncovalent interactions, especially hydrogen bonds [79]. Moreover, benzimidazoles exhibit DNA-binding activity due to their structural similarity to purines [80]. As a result, numerous drugs that have been approved for cancer treatment (bendamustine, liarozole, nocodazole, crenolanib, dovitinib, binimetinib, selumetinib, abemaciclib, pracinostat, galeterone) are benzimidazole-based compounds [79,80]. Benzimidazole–flavonoid hybrids with excellent anticancer activity have been reported in the literature. Nine benzimidazole–chalcone hybrids **143**–**151** (Figure 6), featuring a benzenesulfonamide moiety, demonstrated significant activity (IC_50_ = 0.597–19.995 μM) against HCT-116 colon cancer, MCF-7 breast cancer, and 143B osteosarcoma cells. The benzenesulfonamide moiety was essential for anticancer potency-. In addition, a mono-halogen or mono-methyl group at C3 of the phenyl ring resulted in higher activity than methoxy or trifluoromethyl substitution. It should be mentioned that the positive controls, 5-fluorouracil and odanacatib, were less active than the hybrids. Hybrid **147** was the most active, exhibiting IC_50_ values below 1 μM against all three cancer cell lines (0.597, 0.886, and 0.791 μM, respectively). It also demonstrated significant inhibitory activity on cysteine cathepsins L and K (over 90% inhibition at 5 μM), enzymes associated with cancer progression, through hydrogen-bond interactions with amino acid residues in the enzyme active sites (Gln19, Cys25, Gly68, His163, Gly164 and Gln19, Cys25, Gly64, His162, Trp184, respectively) [81].

EGFR is a transmembrane receptor tyrosine kinase that is frequently overexpressed or mutated in various cancers, including non-small-cell lung cancer, gastrointestinal stromal tumors, colon, and breast cancers. Similarly to imatinib, a selective ATP-competitive inhibitor of EGFR, benzimidazole–chalcone hybrids with an unsubstituted phenyl ring or a *para*-methoxy substituted phenyl ring in the chalcone moiety, **152** and **153** (Figure 6), interacted with amino acids in the binding pocket of EGFR kinase via hydrophobic, electrostatic, and hydrogen-bonding interactions with good docking scores (−8.4 and −8.2 vs. −10.5 KJ/Mol for imatinib). Both hybrids were cytotoxic against HCT-116 colon cancer and H460 non-small-cell lung cancer cells (IC_50_ = 7.31, 12.52 and 10.16, 6.83 μM, respectively), with no observed toxicity toward normal HEK epithelial kidney cells [82].

Topo II, an enzyme that produces transient DNA double-strand breaks, which are crucial for chromosome segregation and cell division, is another key target in anticancer therapy [83]. Topo II inhibitors represent an important class of antitumor agents, including, among others, etoposide and doxorubicin. Benzimidazole–chalcone hybrids have been reported as non-intercalative Topo II catalytic inhibitors. In contrast to Topo II poisons, which stabilize the Topo II–DNA complexes, the catalytic inhibitors block ATP binding to Topo II and inhibit its ATPase activity. Among 21 described hybrids, hybrids **154** and **155** (Figure 6) significantly inhibited the catalytic activity of Topo II (86.9% and 94.9% inhibition at 20 μM, respectively) and suppressed the proliferation of HepG2 hepatoma, A549 lung cancer, LNCaP prostate cancer, and MG63 osteosarcoma cells with IC_50_ values ≤ 5.4 μM. Moreover, in A549 cells, both hybrids strongly inhibited colony formation, being more active than etoposide (IC_50_ = 0.54 and 0.47 μM, respectively, vs. 0.70 μM); reduced migration; and promoted apoptosis. Molecular docking analysis revealed that the hybrids may occupy the ATP-binding pocket of the N-terminal ATPase domain of Topo II—hybrid **154** via two hydrogen bonds with Ser149 through fluor and carbonyl groups, and hybrid **155** via two hydrogen bonds with Asn150 and Ala167 through the carbonyl and methoxy groups of the chalcone unit. For both hybrids, the Surflex-Dock scores exceeded the value obtained for etoposide (6.53 and 9.62 vs. 6.52) [84].

Other benzimidazole–chalcone hybrids have proven to be promising templates for the development of novel therapeutic agents targeting breast and ovarian cancer. Thus, hybrids **156**, **159**, **162**, **163**, **164**, and **166** (Figure 6) showed higher activity than cisplatin on MCF-7 breast cancer cells (IC_50_ = 8.91–11.12 μM vs. 11.70 μM). Hybrids **156**–**161**, **164**–**167**, and **168** demonstrated superior activity compared with cisplatin in OVCAR-3 ovarian cancer cells (IC_50_ = 10.34–14.88 μM vs. 16.04 μM). Hybrid **166** was the most active on both cancer cells. In OVCAR-3 cells, hybrids **156** and **159** blocked the cell cycle at the G2/M phase. The observed cytotoxicity of hybrid **166** in OVCAR-3 cells could be mediated by necrotic mechanisms. Substitution of the benzimidazole moiety with a five- or six-membered N-heterocyclic ring and a hydrocarbon linker was shown to be essential for the observed antitumor activity [85].

An extensive study on the antiproliferative activity of 60 benzimidazole–chalcone and benzimidazole–flavone hybrids revealed that only the former possessed promising antitumor potential, whereas the latter exhibited weak activity. Among benzimidazole–chalcone hybrids, hybrid **160** attracted considerable attention due to its remarkable in vitro and in vivo antitumor activity. Thus, hybrid **160** markedly reduced the proliferation of various cancer cell lines (HepG2, MCF-7, MDA-MB-231, HCT-116, HGC-27, MGC-803, and SGC-7901) with IC_50_ values below 20 μM, whereas normal gastric mucosal cells were less affected (IC_50_ = 34.09 μM). Treatment of HGC-27 and SGC-7901 gastric cancer cell lines with hybrid **160** resulted in the inhibition of colony formation and the induction of apoptosis [86]. Glycolysis, a multistep process involving glucose uptake and its conversion to pyruvate and lactate, is the main source of energy in tumor cells. Given its key role in malignant transformation, tumor growth and invasion, immune evasion, and drug resistance, it has emerged as a therapeutic target in cancer treatment [87]. Hypoxia-inducible factor 1 α (HIF-1α) promotes glycolysis through various mechanisms, including the transcriptional upregulation of key glycolytic enzymes such as hexokinase 2 (HK2), M2-type pyruvate kinase (PKM2), and the platelet isoform of phosphofructokinase (PFKP) [86,88,89]. In HGC-27 cells, hybrid **160** significantly decreased lactate production as well as the expression of HIF-1α, HK2, and PKM2. Moreover, in a subcutaneous xenograft tumor mouse model established with HGC-27 cells, hybrid **160** (15 and 30 mg/kg, every second day for 14 days) reduced tumor growth (inhibitory effects of 30.95% and 62.89%, respectively), induced tumor necrosis, and decreased the expression of HIF-1α and HK2 in tumor tissues. In vivo experiments revealed a favorable safety profile (no mouse deaths, no significant decrease in body weight, no organ damage) and pharmacokinetic profile following intravenous administration [86].

Complex benzimidazole–chalcone hybrids, incorporating 2-quinoline and 1,2,4-thiadiazole moieties, with excellent anticancer activity, have been reported. Hybrids **170**–**174** (Figure 6) exerted notable cytotoxicity on MCF-7 breast cancer, A549 lung cancer, Colo-205 colon cancer, and A2780 ovarian cancer cells (IC_50_ = 0.01–1.99, 0.074–0.88, 0.22–1.58, and 0.083–1.58 μM, respectively) as compared to etoposide, a well-known anticancer drug (IC_50_ = 2.19, 3.34, 0.17, and 1.38 μM, respectively). Hybrids **172** and **173** were the most potent against MCF-7, A549, A2780 and Colo-205 cells, respectively. At the chalcone skeleton, hybrids **170**–**174** incorporated 3,4,5-trimethoxyphenyl, 3,5-dimethoxyphenyl, 4-pyridyl, 2-thienyl, and 3,5-dinitrophenyl moieties; less active hybrids bore 4-methoxyphenyl, 4-nitrophenyl, 4-chlorophenyl, 4-bromophenyl, or 4-(dimethylamino)phenyl moieties [90].

1,3,4-Oxadiazole-containing benzimidazole–chalcone hybrids **175**, **176**, and **177** reduced the proliferation of A549 lung cancer, MCF-7 breast cancer, Panc-1 pancreatic cancer, and HT-29 colon cancer cells with IC_50_ values of 0.95–1.85 μM, comparable to the reference drug doxorubicin (IC_50_ = 0.90–1.41 μM). At 50 μM, the three hybrids did not significantly affect the viability of MCF10A normal cells (91%, 89%, and 94% viability, respectively). Hybrid **175**, featuring an unsubstituted chalcone phenyl ring, exhibited the highest potency across all four cancer cell lines. Substitution of the chalcone phenyl ring with a *para*-chloro atom (**176**) or a *para*-methoxy group (**177**) led to a slight decrease in bioactivity [91]. The three hybrids inhibited BRAF^V600E^ (IC_50_ = 1.70–2.20 μM vs. 0.08 μM for erlotinib), as well as EGFR (IC_50_ = 0.55–0.90 μM vs. 0.08 μM for erlotinib). In addition, in MCF-7 breast cancer cells, the hybrids significantly increased caspase-3, -8, and -9 by 7.2–17 fold; cytochrome c, essential for caspase activation, by 11–14 fold; and pro-apoptotic protein Bax by 31.5–33 fold. Meanwhile, they reduced the anti-apoptotic protein Bcl-2 by 4.9–5.4 fold; these effects were comparable with those of doxorubicin. Hybrid **175** induced accumulation of MCF7 cells in the G0/G1 and S phases, as well as apoptotic cell death. All three hybrids exhibited favorable docking scores within the active sites of EGFR and BRAF^V600E^. In the case of EGFR, they showed the same hydrogen bonds (with Met769 and Asp831) and hydrophobic interactions (with Phe699, Val702, Leu820, and Leu834). For BRAF, the hybrids showed the same hydrophobic interactions (with Ile15, Val23, and Leu66), whereas the hydrogen bonding interactions differed (with Thr81, Cys84, Ser87, and His91 for **175**, Thr81, Cys84, and His91 for **176**, and Thr81 and Cys84 for **177**). The hybrids demonstrated absorption, distribution, metabolism, excretion, and toxicity (ADMET) characteristics comparable to those of erlotinib [91].

Other 1,3,4-oxadiazole-containing benzimidazole–chalcone hybrids **178**–**180** and **181**–**183** were strong inhibitors of EGFR (IC_50_ = 1.8–5.1 μM), while exhibiting moderate to weak activity against BRAF^V600E^. The hybrids demonstrated antiproliferative activity in A549, MCF-7, Panc-1, and HT-29 cancer cells (IC_50_ = 0.80–2.27 μM), with hybrid **179** being the most effective across all four cancer cell lines. Hybrids **178** and **179** induced apoptosis in MCF-7 cells; hybrid **178** also caused cell cycle arrest at the S phase. The hybrids showed higher binding affinities for EGFR compared to erlotinib. Hybrid **179** exhibited two hydrogen bond interactions with Met769 and Thr830 within the EGFR binding site [92].

The p53 tumor suppressor protein restrains the uncontrolled proliferation of mutant cells, and loss of its function is a hallmark of many human cancers. One pathway leading to p53 inactivation involves the oncoprotein hdm2. This protein binds to p53, resulting in its inhibition and degradation via ubiquitination. Imidazolones have been reported as selective inhibitors of hdm2-mediated ubiquitination of p53 [93]. Based on this, imidazolone–chalcone hybrids have been synthesized and tested on 53 tumor cell lines representing nine types of cancer (leukemia, non-small-cell lung, colon, central nervous system, renal, prostate, ovarian, breast, and melanoma). Some of these hybrids, namely **184** and **185** (Figure 6), demonstrated notable anticancer activity, showing GI_50_ values between 1.26 and 10.5 μM. In MCF-7 cells, hybrids **184** and **185** induced cell cycle arrest at the G2/M phase when tested at 10 μM, and at the G0/G1 phase when tested at 30 μM [94].

## 4. Pyrazole–Flavonoid Hybrids

Pyrazole, a five-membered heterocycle consisting of three adjacent carbon atoms and two nitrogen atoms (one “pyrrole-like” and one “pyridine-like”), and its derivatives have demonstrated diverse bioactivities, among which anticancer activity was observed [95]. In cancer cells, pyrazole derivatives act on various targets, including tubulin, CDK, EGFR, vascular endothelial growth factor receptor 2 (VEGFR-2), Bruton’s tyrosine kinase (BTK), and BRAF^V600E^. Thus, the pyrazole scaffold has become an attractive template for the development of novel anticancer drugs. Pyrazofurin is an example of a commercially available drug that contains the pyrazole ring and is used to treat acute and chronic myeloid leukemia [96,97].

**Figure 7:**
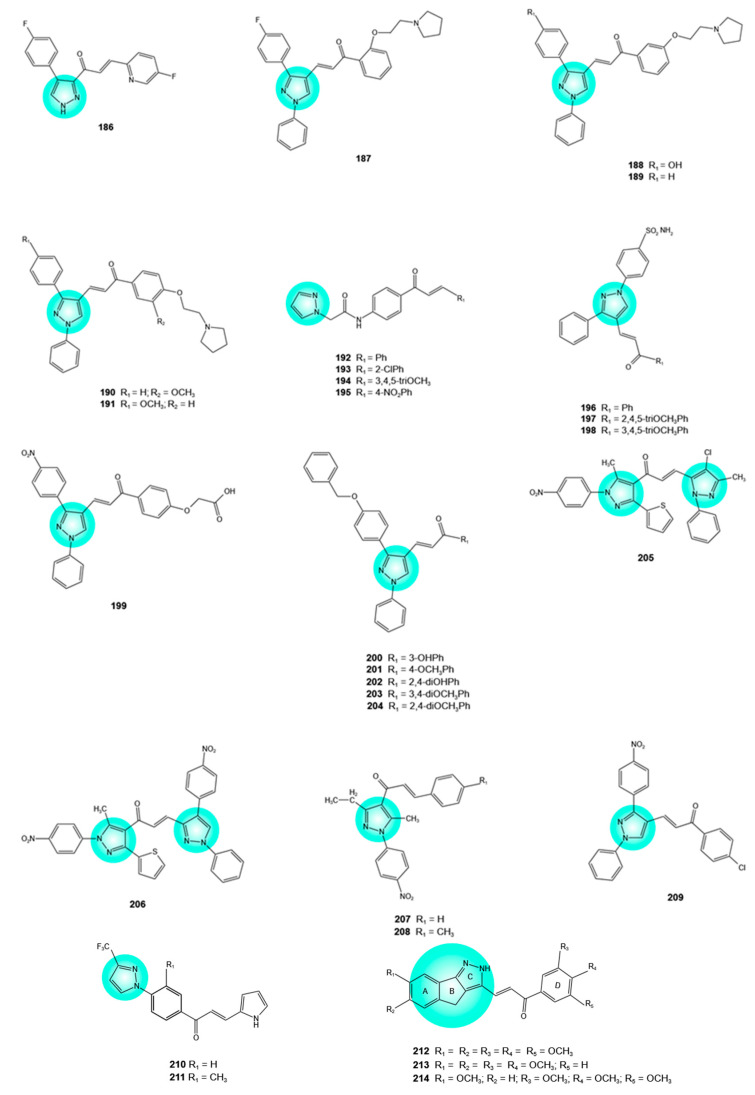
Pyrazole–flavonoid hybrids with antitumor activity.

A pyrazole–chalcone hybrid **186** (Figure 7), bearing 4-fluorophenyl and 5-fluoropyridine moieties, exhibited strong cytotoxicity in MCF-7 breast cancer and HeLa cervical cancer cells (IC_50_ = 0.018 and 0.047 μg/mL, respectively) [23,98].

Other pyrazole–chalcone hybrids with significant activity against MCF-7 cells included hybrids **187**–**191** (Figure 7), which exhibited IC_50_ values ranging from 0.17 to 23.61 μM. Raloxifen and tamoxifen, used as positive controls, displayed significantly lower cytotoxic effects (IC_50_ = 32.66 and 49.81 μM, respectively). All hybrids featured a biphenyl-substituted pyrazole ring and were linked to a pyrrolidine moiety. Further investigations on the most active hybrids, namely **187** and **188**, demonstrated their capacity to induce apoptosis, arrest the cell cycle at the G2/M phase, and decrease the expression of estrogen receptor (ER)-α in MCF-7 cells; the latter effects were slightly lower than those of tamoxifen, a known ER-α protein downregulator (57.78% and 53.63%, respectively vs. 66.40%). Molecular docking analysis revealed similar interactions between hybrids **187** and **188** and the ER-α, as observed for the selective estrogen receptor modulators (SERMs) tamoxifen, 4-OH-tamoxifen, and raloxifen. The phenyl ring on C-3 of pyrazole, along with the pyrrolidinylethoxy and chalcone moieties, contributed to the ER-α affinity of hybrids **187** and **188**. Moreover, molecular dynamics simulation confirmed the stability of the hybrid **187**–ER-α complex. According to Swiss ADME pharmacokinetics predictions, hybrids **187** and **188** possess good oral bioavailability and favorable drug-likeness [99].

Hybrids **192**–**195** (Figure 7) more effectively reduced the viability of MCF-7 breast cancer cells compared with the reference drug, letrozole (IC_50_ = 29.66, 14.66, 17.96, and 15.52 μM, respectively, vs. 30.39 μM). The hybrids showed notable in vitro aromatase inhibitory activity, although they were less potent than letrozole (IC_50_ = 4.65, 1.36, 2.12, and 3.31 μM, respectively, vs. 0.98 μM). Hybrid **193**, the most active cytotoxic agent and aromatase inhibitor, featured a chloro substituent, which appears to be essential for its high bioactivity. According to molecular docking studies, hybrid **193** formed hydrophobic interactions and hydrogen bonds with amino acid residues in the aromatase active site and exhibited strong binding affinity (docking score of −6.215 kcal/mol). In addition, ADME analysis confirmed that all four hybrids have favorable drug-like properties [100].

The pyrazole–chalcone hybrids **196**–**198** (Figure 7) showed remarkable toxicity against oral squamous cell carcinoma cell lines (Ca9-22, HSC-2, HSC-3, and HSC-4), with less potency toward normal oral cells such as gingival fibroblasts, periodontal ligament fibroblasts, and pulp cells (concentration causing 50% cell cytotoxicity (CC_50_) = 1.1–5.2 μM vs. CC_50_ > 340 μM). The three hybrids were selected as the most promising ones from a series of ten derivatives based on their high tumor-selectivity (TS) indices and potency-selectivity expression (PSE) values. Hybrid **197** showed the highest TS and PSE values, followed by **198** and **196**. In HSC-2 cells, hybrids **197** and **198** arrested cell cycle progression at the S and G2/M phases. The trimethoxyphenyl substitution (**197** and **198**) resulted in increased cytotoxicity and improved selectivity. The quantitative structure–activity relationship (QSAR) analysis revealed that TS is positively correlated with three-dimensional structure and lipophilicity [101].

Molecular hybridization of lonazolac, a non-steroidal anti-inflammatory drug containing a pyrazole ring, and a chalcone moiety afforded a series of hybrids among which hybrid **199** (Figure 7) showed the highest potency against HeLa cervical cancer, HCT-116 colon cancer, and RPMI-822 leukemia cells (IC_50_ = 2.41, 2.41, and 3.34 μM, respectively), with weaker effects on MCF-7 breast cancer cells (IC_50_ = 28.93 μM) and MCF10A non-cancer cells (IC_50_ > 50 μM). Since tubulin polymerization is critical for microtubule assembly and cell division, tubulin is a prominent target in anticancer therapy. Hybrid **199** strongly inhibited tubulin polymerization in vitro, being more active than combretastatin A-4, used as a reference drug (IC_50_ = 4.77 vs. 8.143 μM), whereas in HeLa cells, it induced apoptosis and cell cycle arrest at the G2/M phase. Molecular docking studies showed complex interactions between hybrid **199** and various amino acid residues in tubulin, including hydrophobic interactions, three hydrogen bonds, and a metal–acceptor bond [97].

Hybrids **200**–**204** (Figure 7) were found to possess strong cytotoxic activity against MCF-7 breast cancer (IC_50_ = 2.13–7.23 μM), SiHa cervical cancer (IC_50_ = 3.60–5.65 μM), and PC-3 prostate cancer (IC_50_ = 2.97–6.52 μM) cells, with no significant toxicity on HEK293T normal embryonic kidney cells (IC_50_ > 50 μM). Combretastatin A-4 displayed IC_50_ values of 4.12, 5.23, 3.86, and 19.25 μM, respectively. Hybrid **203** exhibited greater potency than combretastatin A-4 against MCF-7 and SiHa cells (IC_50_ values of 2.13 and 3.60 μM, respectively, vs. 4.12 and 5.23 μM, respectively) and surpassed both combretastatin A-4 and vincristine in inhibiting tubulin polymerization (IC_50_ value of 1.15 μM vs. 1.46 and 1.54 μM, respectively). Molecular docking studies revealed hydrogen bond and π-cation interactions between this hybrid and amino acid residues in the colchicine-binding site of tubulin. With respect to SAR, electron-donating groups (hydroxyl, 3,4-dimethoxy, 2,4-dimethoxy, and, to a lesser extent, *para*-methoxy substituents) on the aromatic ring enhanced cytotoxic potency [95,102].

Hybrids **205** and **206** (Figure 7) demonstrated promising activity against breast (MCF-7), prostate (PC-3), and pancreatic (PaCa-2) cancer cells when compared to doxorubicin, a chemotherapeutic drug used prior to surgery whose clinical application is limited by pronounced cardiotoxicity. Hybrid **205** was more effective than doxorubicin in MCF-7 cells (IC_50_ = 42.6 vs. 48.0 μM), whereas hybrid **206** was more active in PaCa-2 cells (IC_50_ = 27.6 vs. 52.1 μM). Unlike doxorubicin, both hybrids showed no cytotoxic effects on BJ1 normal cells. In MCF-7 cells, hybrid **205** downregulated *BCL2* and *CDK4* genes and upregulated the *P21* gene. As Bcl-2 is an antiapoptotic protein, CDK4 regulates cell cycle progression, and p21 is a potent inhibitor of CDKs, these combined effects may lead to apoptosis and cell cycle arrest in MCF-7 cells. In PaCa-2 cells, hybrid **206** downregulated the gene expression of the pro-apoptotic BH3-interacting-domain death agonist (*BID*) and cell cycle progression modulator cyclin D1 (*CCND1*), while upregulating the tumor suppressor *TP53* gene expression. In both cancer cell lines, treatment with hybrids **205** and **206** resulted in DNA damage and fragmentation. Molecular docking studies confirmed the affinity of **205** and **206** for Bcl-2, CDK4, and p53 mutant Y220C, respectively. In addition, numerous interactions between the two hybrids and amino acid residues in the aforementioned proteins were identified: ten interactions between **205** and Bcl-2 (conventional hydrogen bonds, π-cation, alkyl, and π-alkyl interactions), ten interactions between **205** and CDK4 (π-donor hydrogen and ionic bonds, alkyl and π-alkyl interactions), and seven interactions between **206** and p53 mutant Y220C (π-donor hydrogen bonds, π-anion and π-alkyl interactions) [103].

Other hybrids exhibiting higher activity than doxorubicin in PaCa-2 pancreatic cancer cells were hybrids **207** and **208** (Figure 7) (IC_50_ = 13.0 and 24.9 μg/mL, respectively, vs. 28.3 μg/mL). Both hybrids displayed lower cytotoxicity toward BJ1 normal cells (IC_50_ = 74.2 and 81.1 μg/mL, respectively), whereas doxorubicin was more toxic to BJ1 cells than to PaCa-2 cells (IC_50_ = 13.5 vs. 28.3 μg/mL). Treatment of PaCa-2 cells with hybrids **207** or **208** resulted in DNA fragmentation and damage, upregulation of apoptosis-related genes (*CASP3* and *CASP8*, encoding caspase-3 and -8, respectively), and downregulation of the cell proliferation-related gene *CDK6* [104].

Pyrazole–chalcone hybrids with a broader anticancer activity spectrum have been reported. For example, hybrid **209** (Figure 7) demonstrated marked growth inhibition across 37 tumor cell lines, with the greatest sensitivity observed in K-562 leukemia cells (GI_50_ = 0.09 μM) and UO-31 renal cancer cells (CC_50_ = 0.10 μM) [105].

Pyrazole–chalcone hybrids featuring a pyrrole moiety also exhibited broad-spectrum anticancer activity. For example, hybrids **210** and **211** (Figure 7) displayed notable antiproliferative effects across multiple cancer cell lines, including HL-60 and RPMI-8226 (leukemia), KM-12 (colon), BT-549 (breast), OVCAR-3 (ovarian), UO-31 (renal), SNB-75 (central nervous system), and PC-3 (prostate) cells, with IC_50_ values ranging from 0.27 to 2.29 μM, being more active than sunitinib, the reference drug (IC_50_ > 5 μM). The presence of an electron-donating group at the C-3 position of the phenyl ring seems to be critical for antiproliferative activity. A molecular docking analysis indicated that the antiproliferative activity of hybrids **210** and **211** could be attributed to their interaction with VEGFR-2, a receptor tyrosine kinase playing a key role in tumor angiogenesis. Both hybrids **210** and **211**, as well as sunitinib, were found to bind to the ATP-binding site of VEGFR-2. Hybrids **210** and **211** interacted via hydrogen bonds, hydrophobic, and van der Waals interactions, whereas sunitinib primarily formed hydrogen bonds. In silico ADME studies of hybrids **210** and **211** indicated favorable drug-likeness [106].

Other hybrids, including an indenopyrazole moiety, have been reported as potent inhibitors of the EGFR/protein kinase B (Akt) pathway, which is involved in tumor promotion and metastasis. Hybrids **212**–**214** (Figure 7), at only 10 μM, inhibited the EGFR/Akt pathway in A549 lung cancer cells, along with a significant reduction in cell growth and proliferation. According to molecular docking studies, the three hybrids bind to the EGFR tyrosine kinase and exhibit higher interaction stability and binding affinity compared with erlotinib. In addition, in A549 cells, hybrids **212**–**214** induced cell cycle arrest at the G2/M phase, upregulated the tumor suppressor protein p53 and the pro-apoptotic protein Bax, and downregulated the anti-apoptotic protein Bcl-2. The hybrids exhibited cytotoxic effects not only against A549 cells but also against MCF-7 breast cancer, MDA-MB-231 triple-negative breast cancer, HCT-116 colon cancer, and SKBR3 breast cancer cells (IC_50_ values < 7 μM), with weaker toxicity on HEK293T normal cells (IC_50_ = 32–46 μM). All three hybrids showed greater potency than erlotinib against A549, HCT-116, and MCF-7 cells. Cytotoxic activity depends on methoxy substituents on the A and D rings, with two or three methoxy groups on the D ring markedly enhancing potency [107].

## 5. Isoxazole–Flavonoid Hybrids

The structural characteristics of isoxazole, a five-membered heterocycle containing nitrogen and oxygen at adjacent positions, enable various noncovalent interactions (hydrogen bonding, π-π stacking, hydrophilic interactions), which support its binding to a broad range of protein targets. Isoxazole-containing compounds are known for their broad spectrum of activity, including anticancer effects. Tivozanib, a drug used to treat renal cell carcinoma, contains the isoxazole moiety [108,109]. The hybridization of the isoxazole moiety with the flavonoid ring has been extensively explored in recent years to enhance or broaden antitumor activity and reduce toxicity. Numerous isoxazole–flavonoid hybrids promising for cancer therapy have been synthesized and characterized.

**Figure 8:**
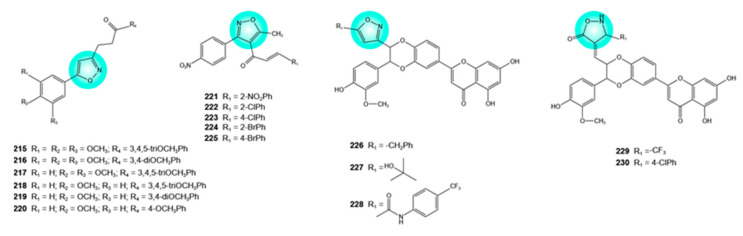
Isoxazole–flavonoid hybrids with antitumor activity.

Several isoxazole–chalcone hybrids **215**–**220** (Figure 8) with significant cytotoxic activity against DU-145 prostate cancer cells (IC_50_ = 0.96–1.93 μM) have been reported. The hybrids also exhibited cytotoxic activity against MCF-7 and MDA-MB-231 breast cancer cells and A549 lung cancer cells (IC_50_ = 2.18–6.35 μM). The anticancer potential was associated with the presence of methoxy substituents on the phenyl rings [110].

Isoxazole–chalcone hybrids **221**–**225** (Figure 8) showed growth inhibitory effects on several non-small-cell lung cancer cells (H1792, H157, A549, and Calu-1), with **222** and **223** showing the highest potency. Hybrid **222** inhibited the growth of H157, A549, and Calu-1 cells with IC_50_ values of 1.35, 1.48, and 2.07 μM, respectively, whereas hybrid **224** displayed IC_50_ values below 10 μM against H157 and Calu-1 cells (7.27 and 8.98 μM, respectively). An electron-withdrawing group at the C-2 position of the phenyl group in the aryl moiety was more favorable for the antiproliferative activity than substitution at the C-4 position. In A549 cells, all hybrids induced apoptosis via the upregulation of death receptor 5 and activation of caspase-8, -9, and -3, as well as PARP cleavage [111].

Hybrids including hydnocarpin, a flavonolignan isolated from the seeds of *Hydnocarpus wightiana* Blume, and isoxazole/isoxazolone moieties, are notable for their activity against A549 lung cancer cells and A375 melanoma cells. IC_50_ values below 1 μM were determined for hybrids **226**–**230** (Figure 8) against A375 cells, and for hybrid **228** against A549 cells; hybrid **230** showed an IC_50_ of only 1.2 μM against A375 cells. All the aforementioned hybrids showed lower toxicity on WI38 lung fibroblast cells (normal cells). In A549 and A375 cells, hybrids **228** and 200 triggered apoptosis through activation of the caspase pathway [112].

## 6. Thiazole–Flavonoid Hybrids

The thiazole ring, a five-membered aromatic heterocycle containing sulfur and nitrogen atoms, is endowed with potent antitumor activity achieved through various mechanisms, including the inhibition of enzymes involved in carcinogenesis, DNA intercalation, the induction of apoptosis, and the inhibition of angiogenesis, fibrosis, nuclear factor kappa-light-chain-enhancer of activated B cells (NF-κB), phosphatidylinositol-4,5-bisphosphate 3-kinase/mechanistic target of rapamycin (PI3K/mTOR), and ribosomal S6 kinase 2 (RSK2). Clinically used anticancer drugs, including tiazofurin, dasatinib, dabrafenib, ixabepilone, epothilone, and patellamide A, bear a thiazole scaffold. Moreover, natural thiazole-containing cyclic peptides of bacterial origin, such as berninamycin, micrococcin, and thiocillin I, represent promising candidates for cancer treatment [113].

**Figure 9:**
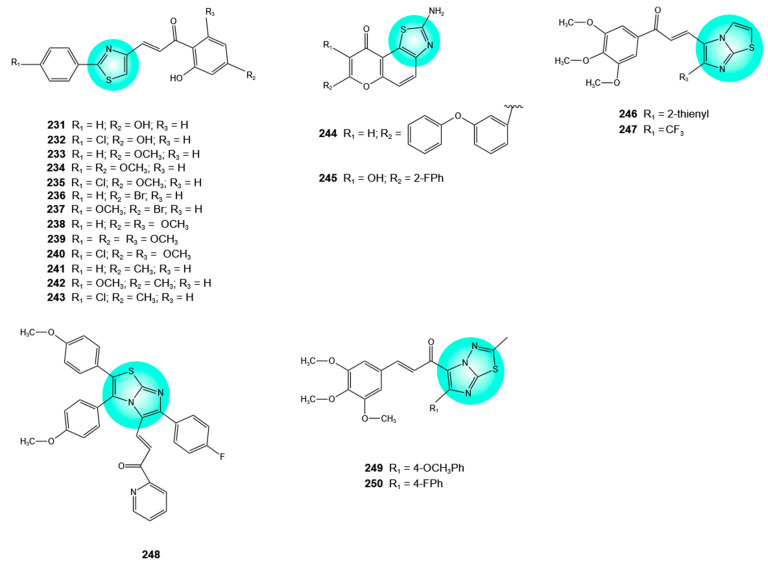
Thiazole–flavonoid hybrids with antitumor activity.

Thiazole–*ortho*-hydroxychalcone hybrids **231**–**243** (Figure 9) exhibited cytotoxic effects in both sensitive (CCRF-CEM leukemia, MDA-MB-231 breast cancer, HCT116(p53^+/+^) colon cancer, U87MG glioblastoma, and HepG2 hepatocarcinoma cells) and resistant (CEM/ADR5000 leukemia, MDA-MB-231/BCRP breast cancer, HCT116(p53^−/−^) colon cancer, and U87MG/ΔEGFR glioblastoma cells) cancer cell lines [114]. Resistance in these cancer cell lines is mediated through various mechanisms: overexpression of P-gp (CEM/ADR5000 cells), overexpression of BCRP (MDA-MB-231/BCRP cells), deletion or inactivation of *TP53* tumor suppressor gene (HCT116(p53^−/−^) cells), and expression of a mutant form of EGFR (U87MG/ΔEGFR cells) [114,115]. All hybrids showed IC_50_ values below 10 μM against CEM/ADR5000 and HCT116p53^−/−^ cells, while selected hybrids **232**, **237**, **238**, and **243** and **235**, **237**, **238**, **241**, **242**, **243** were similarly active against MDA-MB-231/BCRP and U87MG/ΔEGFR cells, respectively. These thiazole–*ortho*-hydroxychalcone hybrids emerge as potential agents for overcoming drug resistance in cancer [114].

Two 2-aminothiazole–flavonoid hybrids, **244** and **245** (Figure 9), reduced the viability of glioblastoma (U87, U251, U138, T98G) and neuroblastoma (SK-N-SH) cells with IC_50_ values of 1.2–2.8 μM. Structural moieties, such as a diphenyl ether group in **244**, a hydroxyl group at C-3 of ring C and a fluoro substituent on ring B in **245**, were found to play a major role in the high bioactivity. Both derivatives strongly interact with Tau protein, an emerging anticancer target in glioblastoma multiforme, one of the most aggressive and lethal brain tumors [116]. Tau protein, known for its crucial role in the assembly and stabilization of the microtubule cytoskeleton, is also involved in glioblastoma progression by modulating 3D cell organization and functions via the N-cadherin-PI3K/Akt signaling axis [116,117]. In U87 cells expressing the Tau protein (U87 shCTRL cells), hybrids **244** and **245** caused alterations in the mitochondrial network and impaired cell migration through remodeling of the microtubule cytoskeleton. The previously mentioned effects were not observed in U87 cells not expressing the Tau protein (U87 shTau cells) [116]. In multi-cellular spheroids, a 3D human cell model that mimics glioblastoma multiforme organization, the two hybrids reduced cell dissemination from spheroids formed by U87 shCTRL cells, with no significant effect on spheroids formed by U87 shTau cells. Moreover, hybrid **245** (single and multiple treatments) significantly reduced the growth of spheroids formed by U87 shCTRL cells. These latest findings demonstrate that hybrids **244** and **245** target the Tau protein in glioblastoma cells, reducing Tau-dependent cell migration (both hybrids) and proliferation (hybrid **245**) [118]. Each of the two hybrids is a promising candidate for the development of drugs targeting glioblastoma multiforme [116,118].

Hybrids incorporating chalcone and imidazothiazole moieties and exhibiting significant, broad-spectrum anticancer activity have been reported. Hybrids **246** and **247** (Figure 9) were cytotoxic to MCF-7 cells at concentrations as low as 4 μM. In MCF-7 cells, both hybrids induced cell cycle arrest at the G0/G1 phase, downregulated cyclins D1 and E1, upregulated CDK4, tumor suppressor proteins (p53, p21, and chk2), and caspase-9, and suppressed NF-κB [119].

Hybrid **248** (Figure 9), bearing a pyridyl ring, reduced the proliferation of MCF-7 breast cancer, A549 lung cancer, HeLa cervical cancer, DU-145 prostate cancer, and HT-29 colorectal cells with IC_50_ values ranging from 0.64 to 1.44 μM. In A549 cells, the hybrid inhibited tubulin polymerization, induced cell cycle arrest at the G2/M phase, and induced apoptosis. Molecular docking experiments demonstrated the binding of the hybrid to tubulin at the colchicine binding site [120].

Two imidazothiadiazole–chalcone hybrids, **249** and **250** (Figure 9), showed high cytotoxicity against DU-145 prostate cancer, MDA-MB-231triple-negative breast cancer, MCF-7 breast cancer, and A549 lung cancer cells (IC_50_ = 0.65–2.25 μM.). In DU-145 cells, the hybrids induced G0/G1 phase cell cycle arrest, activated caspases 3 and 8, downregulated cyclin D1, and upregulated p21 and p27 CDK inhibitors. The presence of 3,4,5-trimethoxy groups in both hybrids correlated with their potent cytotoxic activity [121].

## 7. Concluding Remarks

This review provides an overview of azole–flavonoid hybrids that are promising candidates for anticancer drug development. The combination of azole and flavonoid moieties afforded hybrids with enhanced antitumor potency and selectivity. Most reported hybrids demonstrated cytotoxicity across a panel of cancer cell lines. Others were tested and were active against specific cancer cell lines, such as PC-3 prostate cancer cells (**16**–**19**, **121**), A549 lung cancer cells (**20**–**26**), MCF-7 breast cancer cells (**89**–**91**, **101**, **187**–**191**, **246**, **247**), HeLa cervical cancer cells (**88**), MGC-803 gastric cancer cells (**95**, **96**), RPMI-8226 (**1**–**4**) and CCRF-CEM (**67**–**73**) leukemia cells. In particular, hybrid **92** exhibited cytotoxicity against various ovarian cancer cell lines, hybrids **93** and **94** were active against cervical cancer cells, hybrids **196**–**198** against oral cancer cells, and hybrid **27** against liver cancer cells, while hybrids **244** and **245** displayed activity against glioblastoma and neuroblastoma cells. Some azole–flavonoid hybrids exhibited remarkable antitumor activity, being active in the nanomolar range (e.g., **1**, **2**, **10**–**14**, **82**–**87**, **113**–**121**, **147**, **248**) and/or being more potent than the reference drugs (e.g., **1**, **2**, **5**, **15**, **20**–**24**, **36**–**47**, **93**–**97**, **156**–**168**). Specific structural features played a critical role in modulating the anticancer potential of azole–flavonoid hybrids. Key SAR aspects of azole–flavonoid hybrids are summarized in Table A1.

Combining azole and flavonoid moieties has yielded hybrids with multitarget anticancer activity. For instance, hybrids **175**–**177** acted on BRAF^V600E^, EGFR, cytochrome c, Bax, and Bcl-2. Several other hybrids (**130**–**132**, **160**, **205**–**208**, **212**–**214**, **246**, **247**, **249**, and **250**) also displayed multitarget activity.

Notably, numerous hybrids displayed tumor-selective cytotoxicity, with minimal impact on normal cells. This ability to selectively kill cancer cells may partly result from off-target interactions. Protein kinases, as key regulators of the cell cycle, proliferation, and apoptosis, represent important targets in cancer therapy. Many kinase inhibitors (imatinib, dasatinib, sorafenib, gefitinib, and axitinib) also act on non-kinase off-targets (reduced nicotinamide adenine dinucleotide phosphate (NADPH) quinone oxidoreductase 2, transthyretin, phosphodiesterase 6D, mitochondrial dihydroorotate dehydrogenase, E3 ubiquitin-protein ligase), which can significantly contribute to their efficacy [122]. Similarly, the selective cytotoxicity of certain azole–flavonoid hybrids could arise from off-target activity. Although we are not aware of such studies, this mechanism cannot be excluded, and the potential role of off-target activity in both bioactivity and toxicity should be taken into consideration and further investigated.

Several hybrids exhibited cytotoxicity against MDA-MB-231 (**28**–**34**, **36**–**47**, **84**–**86**, **105**, **106**, **113**–**121**, **128**, **129**) and MDA-MB-468 (**60**–**62**) triple-negative breast cancer cells, which serve as models for aggressive and chemoresistant tumors. Other hybrids exhibited cytotoxicity in other MDR cancer cells: **65** and **66** in NCI-H460/R non-small-cell lung carcinoma, DLD1-TxR colorectal carcinoma, and U87-TxR glioblastoma cells, and **231**–**243** in CEM/ADR5000 leukemia, HCT116(p53^−/−^) colon cancer, and U87MG/ΔEGFR glioblastoma cells.

Although azole–flavonoid hybrids have demonstrated promising anticancer activity in cell-based assays, key challenges remain, including pharmacokinetic profile, in vivo efficacy and toxicity, and the development of drug resistance.

The pharmacokinetic profiles of azole–flavonoid hybrids have been predicted by in silico ADME studies. Although certain hybrids (**133**–**136**, **175**–**176**, **187**, **188**, **210**, **211**) showed acceptable ADME properties, in vivo pharmacokinetic investigations are essential to confirm systemic bioavailability. The azole moiety increases the lipophilicity (logP) of the hybrids, thereby promoting cellular membrane penetration and tissue accumulation, both of which contribute to bioavailability and therapeutic efficacy [6,101]. However, in vivo confirmation of these benefits is still required.

The in vivo efficacy and toxicity of azole–flavonoid hybrids remain largely unexplored. To the best of our knowledge, animal studies are scarce, and no studies involving human subjects have been conducted. Potential toxicity represents a limitation for the therapeutic application of azole–flavonoid hybrids, since both azoles and flavonoids possess toxic potential. Prolonged use of azole antifungals has been associated with hepatotoxicity, hormone-related adverse effects (oligospermia, azoospermia, gynecomastia, impotence, hyponatremia, hypokalemia, and adrenal insufficiency), alopecia, myositis, and peripheral neuropathies [123]. Although widely considered safe, flavonoids can induce carcinogenicity, as well as liver, renal, and thyroid toxicity, endocrine dysfunction, and disturbances of gut microbiota. All these adverse health effects are dose- and structure-dependent [124,125].

Another critical challenge is the potential development of resistance during long-term administration of azole–flavonoid hybrids. It is well-known that fungal pathogens develop resistance to azoles through various mechanisms, including alteration or overexpression of drug targets, efflux pump expression, genomic alterations, cellular stress responses, and epigenetic regulation [125,126]. The emergence of azole resistance in fungi provides a basis for understanding how resistance may arise in cancer cells exposed to azole–flavonoid hybrids. The design of azole–flavonoid hybrids and dose optimization are important strategies to overcome the potential development of resistance in tumor cells.

Synthetic scalability is also critical for the future development of azole–flavonoid hybrids. Although hybrid synthesis was not covered in the present review, it should nevertheless be noted that the synthetic routes generally involve multiple steps, result in low yields, and require long reaction times, making large-scale synthesis challenging. Other limitations include the generation of secondary products and the use of toxic reagents [33,34,35,36,53,54,55,56,57,58,59,97,98,99,100,102,103,104,105,106,107]. These drawbacks could be addressed by optimizing synthetic routes and using green methodologies.

Despite the large number of cell-based studies demonstrating the anticancer potential of azole–flavonoid hybrids, patent activity is limited. Our patent search identified only one patent describing triazole–flavonoid hybrids capable of reversing MDR in cancer cells through the inhibition of P-gp, MRP1, and BCRP [127].

Taken together, this review highlights the potential of azole–flavonoid hybrids as promising candidates for anticancer drug development. Although the azole–flavonoid hybrids reviewed in the present work exhibit several advantages (nanomolar-range activity, multitarget cytotoxic activity, selective tumor cytotoxicity, and cytotoxicity against MDR cancer cells), animal and human studies are required to confirm their efficacy and safety in vivo.

## Data Availability

No new data were created or analyzed in this study.

## Abbreviations

The following abbreviations are used in this manuscript:

FOLFOX	Folinic acid, 5-fluorouracil, and oxaliplatin
FLAM	Flavopiridol, cytarabine, and mitoxantrone
SAR	Structure–activity relationship
BET	Bromodomain and extraterminal
PARP 1	Poly(ADP-ribose) polymerase 1
IC_50_	Half maximal inhibitory concentration
Bax	Bcl-2 associated X protein
Bcl-2	B-cell lymphoma 2
Topo IIα	Topoisomerase IIα
GI_50_	Concentration causing 50% growth inhibition
EGFR	Epidermal growth factor receptor
LD_50_	Concentration causing 50% cell death
MDR	Multidrug-resistant/resistance
P-gp	P-glycoprotein
ABC	ATP-binding cassette
BCR-ABL	Breakpoint Cluster Region–Abelson
CDK4, 6, and 9	Cyclin-dependent kinase 4, 6, and 9
PROTAC	Proteolysis Targeting Chimera
MRP1	Multidrug-resistance-associated protein 1
BCRP	Breast cancer resistance protein
EC_50_	Concentration producing 50% of the maximal effect
ADME	Absorption Distribution Metabolism Excretion
ADMET	Absorption Distribution Metabolism Excretion Toxicity
HIF-1α	Hypoxia inducible factor 1 α
HK2	Hexokinase 2
PKM2	M2-type pyruvate kinase
PFKP	Phosphofructokinase
VEGFR-2	Vascular endothelial growth factor receptor 2
BTK	Bruton’s tyrosine kinase
ER	Estrogen receptor
SERM	Selective estrogen receptor modulator
CC_50_	Concentration causing 50% cell cytotoxicity
TS	Tumor selectivity
PSE	Potency-selectivity expression
QSAR	Quantitative structure–activity relationship
Akt	Protein kinase B
BID	BH3-interacting-domain death agonist
CCND1	Cell cycle progression modulator cyclin D1
NADPH	Reduced nicotinamide adenine dinucleotide phosphate
NF-κB	Nuclear factor kappa-light-chain-enhancer of activated B cells
PI3K/mTOR	Phosphatidylinositol-4,5-bisphosphate 3-kinase/mechanistic target of rapamycin
RSK2	Ribosomal S6 kinase 2

## Appendix A

**Table A1:** Key SAR aspects of azole–flavonoid hybrids.

Hybrid	Type of Hybrid	Structural Features Enhancing Anticancer Activity	Cancer Cell Lines	References
	1,2,3-triazole–chalcone	bromophenyl group at N-1 of triazole *meta*-methoxy group on chalcone phenyl ring	RPMI-8226	[24]
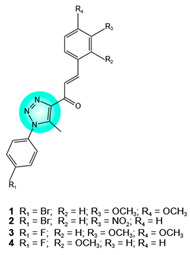
	1,2,3-triazole–chalcone	unhalogenated benzyl group at N-1 of triazole	MCF-7 MIA-PaCa-2 A549 HepG2	[25]
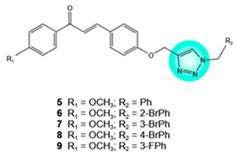
	1,2,3-triazole–chalcone (flavokawain B-based chalcone hybrids)	3,4-dimethoxyphenyl chalcone moiety	RPMI-8226 SR, K-562 M14 MCF-7 HCT-116	[26]
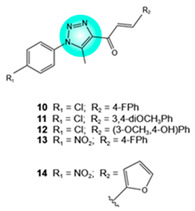
	1,2,4-triazole–chalcone	allyl-substituted triazole *para*-chloro or *para*-methoxy substitution on the phenyl ring	A549	[29]
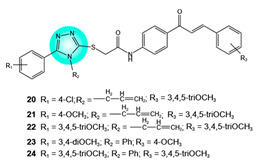
	1,2,4-triazole–chalcone	methoxy group on the chalcone B ring *para*-halophenyl substitution at N-1 of the triazole unit (F or Cl)	A549	[19,30]
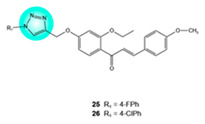
	1,2,3-triazole–chalcone	3,4-dimethoxybenzene A ring in the chalcone moiety *meta*-chloro substituent on the benzene ring	MCF-7 MDA-MB-231	[19,33]
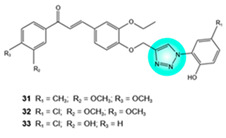
	1,2,3-triazole-benzimidazole– chalcone	benzyl substitution at N-1 of triazole chloro substitution at chalcone moiety tri-pharmacophore structure	PC-3 T47-D MDA-MB-231	[19,34]
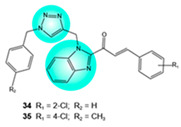
	1,2,4-triazole tethered quinoline–chalcone	allyl-triazole moiety chloro and 3,4,5-trimethoxy groups on the 3-phenyl acryloyl ring unsubstituted phenyl ring attached to quinoline	PANC-1 MCF-7 HT-29 A549	[38]
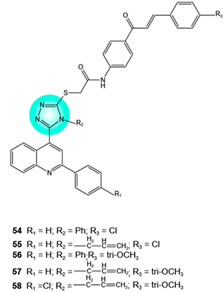
	1,2,3-triazole ferrocenyl chalcone-quinine/quinidine	quinidine moiety tri-pharmacophore architecture (ferrocene + chalcone + cinchona alkaloid)	NCI-H460 NCI-H460/R DLD1 DLD1-TxR U87 U87-TxR	[19,43]
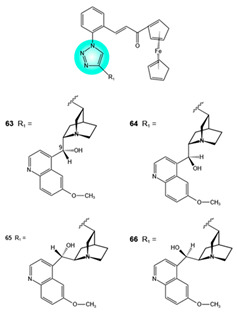
	1,2,3-triazole ferrocenyl –chalcone–uracil	ferrocenyl chalcone scaffold long alkyl chains (*n* = 5, 6, 8) uracil moiety	CCRF-CEM	[19,48]
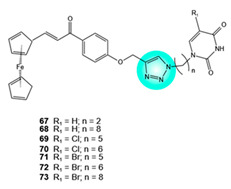
	1,2,3-triazole–flavone	electron-withdrawing substituent on the phenyl ring linked to triazole	MDA-MB-231 IMR-32	[19,53]
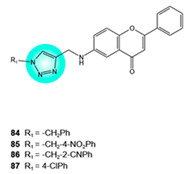
	1,2,3-triazole–flavone– benzimidazole	electron-withdrawing group at *para* (C-4) position of the benzene ring unsubstituted flavone scaffold	MCF-7	[19,55]
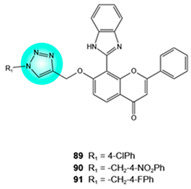
	1,2,3-triazole–flavonol	halogen group on R_1_ trifluoromethyl group on the benzene ring a substituted phenyl ring attached to the triazole moiety	HCT-116 OVCAR-3	[19,66]
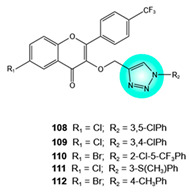
	1,2,3-triazole–isoflavone	phenyl ring attached to the 1,2,3-triazole unit methoxy substitution on the phenyl ring linked to the triazole	PC3 DU-145 MCF-7 A549	[68]
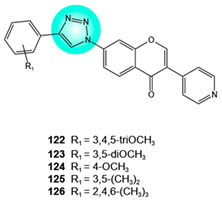
	benzimidazole–chalcone	benzenesulfonamide moiety mono-halogen substitution at C-3 of the phenyl ring mono-methyl substitution at C-3 of the phenyl ring	HCT-116 MCF-7 143B	[81]
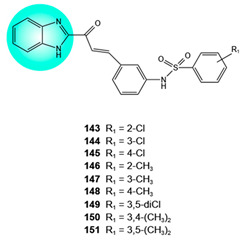
	benzimidazole–chalcone	multi-pharmacophore architecture (incorporating 2-quinoline and 1,2,4-thiadiazole moieties) substituents on the chalcone phenyl ring (3,4,5-trimethoxyphenyl, 3,5-dimethoxyphenyl, 4-pyridyl, 2-thienyl, 3,5-dinitrophenyl)	MCF-7 A549 Colo-205 A2780	[90]
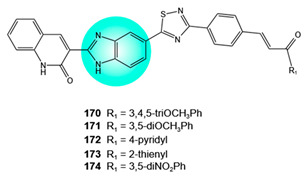
	pyrazole–chalcone	phenyl ring at C-3 of pyrazole pyrrolidinylethoxy substituent on chalcone moiety	MCF-7	[99]
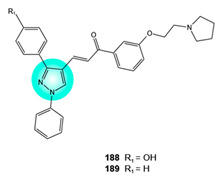
	indenopyrazole–chalcone	methoxy substituents on the A and D aromatic rings two or three methoxy groups on the D ring indenopyrazole moiety	A549 MCF-7 MDA-MB-231 HCT-116 SKBR3	[107]
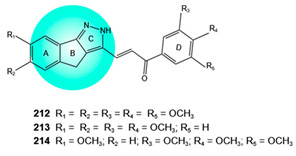
	isoxazole–chalcone	aryl (phenyl) ring attached to the chalcone moiety electron-withdrawing substituent at the C-2 (*ortho*) position of the phenyl ring C-2 (*ortho)* substitution favored over C-4 (*para*) substitution	H1792 H157 A549 Calu-1	[111]
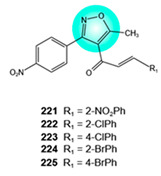
	2-aminothiazole–flavonoid	diphenyl ether moiety hydroxyl group at C-3 of ring C fluoro substituent on ring B	U87 U251 U138 T98G SK-N-SH	[116]
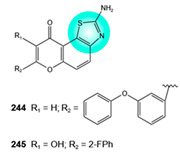
	imidazothiadiazole–chalcone	3,4,5-trimethoxy substitution on the aromatic ring	DU-145 MDA-MB-231 MCF-7 A549	[121]
